# Novel ERP Evidence for Processing Differences Between Negative and Positive Polarity Items in German

**DOI:** 10.3389/fpsyg.2019.00376

**Published:** 2019-03-06

**Authors:** Mingya Liu, Peter König, Jutta L. Mueller

**Affiliations:** ^1^Institute of Cognitive Science, Osnabrück University, Osnabrück, Germany; ^2^Department of Neurophysiology and Pathophysiology, University Medical Center Hamburg-Eppendorf, Hamburg, Germany

**Keywords:** polarity item, negation, ERP, German, syntax-related P600, pragmatics-related P600

## Abstract

One unresolved question about polarity sensitivity in theoretical linguistics concerns whether and to what extent negative and positive polarity items are parallel. Using event-related brain potentials (ERPs), previous studies found N400 and/or P600 components for negative and positive polarity violations with inconsistent results. We report on an ERP study of German polarity items. Both negative and positive polarity violations elicited biphasic N400/P600 effects relative to correct polarity conditions. Furthermore, negative polarity violations elicited a P600-only effect relative to positive polarity violations. The lack of a graded N400 effect indicates that both kinds of violations involve similar semantic processing costs. We attribute the increase in P600 amplitude of negative polarity violations relative to positive polarity violations to their different nature: the former are syntactic anomalies triggering structural reanalysis, whereas the latter are pragmatic oddities inducing discourse reanalysis. We conclude that negative and positive polarity violations involve at least partly distinct mechanisms.

## Introduction

How individual words are used and understood in a sentence depends on the narrow linguistic context (i.e., syntactic and semantic properties of the sentence) as well as on the broad pragmatic context (i.e., properties of the discourse where the sentence is embedded). A prototypical showcase for the effect of context in sentence processing is the phenomenon of polarity sensitivity. Negative polarity items (NPIs), such as English *ever*, tend to occur only in negative contexts. For example, in the list below (1a) is considered improper, but (1b) is a well-formed sentence. In close analogy, positive polarity items (PPIs) such as English *already* tend only to occur in affirmative contexts. (1c) is a well-formed sentence, but the negated sentence (1d) is odd. In contrast, words such as English *really* are polarity-insensitive items (henceforth, non-PIs), as they can occur in affirmative as well as negative contexts, e.g., (1e)/(1f). Thus, there is a close interaction of the usage of specific words and the positive or negative polarity of the context. These differences between NPIs, PPIs and non-PIs have been confirmed in behavioral studies (e.g., Liu and Soehn, 2009; Richter and Radó, 2013) in terms of acceptability or naturalness ratings, which are widely used in psycholinguistics (e.g., Masia et al., 2017).

(1) (a) *This has ever been done before.*
  (b) *This hasn’t ever been done before.*
  (c) *This has already been done before.*
  (d) *This hasn’t already been done before.*
  (e) *This has really been done before.*
  (f) *This hasn’t really been done before.*

Polarity sensitivity has been a key field of research in generative linguistics. It is revealing regarding the internal structure of language, i.e., how different aspects of grammar and pragmatics interact with one another. Most of the theoretical literature attempts to characterize the properties of the contexts in which polarity items can or cannot occur from a syntactic or semantic point of view. Klima (1964) proposes a syntactic generalization that English NPIs such as *ever* and *anything* are acceptable (i.e., licensed) only if they are in the syntactic scope of negation or negation-like contexts. For example, *not* can license the NPI *any* in its scope, as in *Tom doesn’t like any city*. By contrast, *Tom doesn’t like cities but Mary likes any city* is ungrammatical despite the presence of negation as the NPI is not in the scope of negation. This view dominated the field for many years.

However, Ladusaw (1979) points out the limits of Klima’s syntactic account. He observes that not only negative quantifiers such as *no*, but also positive quantifiers such as *every* license NPIs. “*No/Every student who had ever read anything about phrenology attended the lecture*” may serve as an example. For such reasons, Ladusaw proposes a logico-semantic generalization that an NPI is acceptable only if it is interpreted in the semantic scope of a downward monotonic^1^ expression. Both *every* and *no* are downward monotonic in their first argument position (i.e., the modified head noun plus the relative clause), and thus license NPIs there. Alternative semantic accounts have emerged since the 1990s (e.g., Kadmon and Landman, 1993; Krifka, 1995; Giannakidou, 1998; Chierchia, 2004, 2006). Giannakidou (1998), for instance, proposes that an NPI is acceptable only if it is interpreted in the semantic scope of a non-veridical^2^ (i.e., not-truth-preserving) expression. As downward monotonic expressions are a subset of non-veridical contexts, this extension covers additionally those linguistic contexts that are not or not straightforwardly downward monotonic, such as questions (e.g., “*Did Bill buy anything?*”) or conditionals (e.g., “*If this has ever been done before, we need to come up with an alternative plan*”). In addition to syntax and semantics, pragmatic aspects related to NPIs have also been studied. Kadmon and Landman (1993), for instance, argue that licensed NPIs create a strengthening effect of the statement. In a recent paper, Liu (2019) reports on experimental evidence that licensed NPIs in conditionals express a lower degree of speaker credence toward the antecedent (e.g., *if this has ever been done before* vs. *if this has been done before*). We leave these pragmatic aspects aside because they address the interpretive effects of licensed NPIs and are thus less relevant for the licensing question of NPIs. Focusing on the latter, we assume that sentences with unlicensed NPIs such as “*This has ever been done before*” are treated differently by the accounts reviewed above. According to Klima (1964), they are syntactic anomalies due to the lack of negation in structure. In contrast, Ladusaw (1979) and Giannakidou (1998) assume that they are semantic anomalies due to the lack of negation in meaning.

While semantic accounts of NPIs have been dominating the field since Ladusaw’s work, a purely semantic approach cannot be adopted because NPIs must be in a certain structural (i.e., c-command) relation with a licenser so that they lie in its semantic scope. In other words, we need syntax to compute semantic scope. For such reasons, an integrative approach combining syntax and semantics is more desirable. Chierchia (2004, 2006), for instance, models NPI licensing by combining alternative semantics and a feature-checking mechanism in syntax. Following this, or by integrating the syntactic view by Klima (1964) with the semantic view by e.g., Giannakidou (1998), we can assume that sentences with unlicensed NPIs are syntactic and semantic anomalies.

While PPIs were taken to be less interesting than NPIs in earlier literature (e.g., Horn, 1989), they have been gaining more attention recently (e.g., Szabolcsi, 2004; Homer, 2011; Liu, 2017; Nicolae, 2017; Zeijstra, 2017; Hoeksema, 2018). Theoretical accounts of PPIs are, however, highly biased by NPI theories, in that they often assume a parallelism between NPIs and PPIs. For instance, Progovac (1994) proposes that a PPI is unacceptable (i.e., anti-licensed) in the syntactic scope of negation in contrast to NPIs. Alternatively, some researchers hold that PPIs are unacceptable in the semantic scope of downward monotonic or non-veridical expressions (van der Wouden, 1997; Giannakidou, 1998, 2012; Csipak et al., 2013). Liu and Soehn (2009) report on the results of a behavioral study in German, showing that NPIs without negation (such as “*This has ever been done before.*”) and PPIs with negation (such as “*This hasn’t already been done before”*) received equally low acceptability ratings. These studies by and large hint that PPIs are parallel to NPIs in that they are oppositionally sensitive to negation or negation-related contexts: sentences with anti-licensed PPIs are as anomalous as unlicensed NPIs. To briefly clarify, the terms of unlicensed NPIs and anti-licensed PPIs are from theoretical linguistics. NPIs without negation are unlicensed due to the lack of a licensor, whereas PPIs with negation are anti-licensed due to the presence of an anti-licensor, that is, negation that renders the PPI odd. Henceforth, we will use these consistently throughout the paper for NPI vs. PPI violations respectively.

However, the assumption about NPIs and PPIs being parallel has been under debate. Do they share similar licensing conditions? Are NPI and PPI violations anomalous to the same degree and for the same (i.e., syntactic and semantic) reasons (see Iordachioaia and Liu, 2018; Liu and Iordachioaia, 2018)? Szabolcsi (2004) observes that PPIs are not sensitive to downward monotonic contexts but to anti-additive^3^ contexts. For example, the PPI *something* is fine with the downward monotonic quantifier *few* but not with the anti-additive quantifier *nobody*. Thus, the sentence “*Few people ate something”* is fine, but *“Nobody ate something*” is bad.

Based on theoretical considerations, Liu (2017) argues that unlicensed NPIs (e.g., 1a), i.e., due to the absence of negation, are ungrammatical and not repairable. In contrast, anti-licensed PPIs (e.g., 1d), i.e., due to the presence of negation, are often only pragmatically odd and therefore repairable by enriched context, as in (2). Thus, according to Liu (2017), NPIs and PPIs differ principally.

(2) A: *John already came.*
  B: *He did* ***not already*** *come. In fact, he is quite late.*

In summary, it remains an open question whether and to what extent NPIs and PPIs are parallel. In other words, whether their requirements on context and violations of these (e.g., unlicensed NPIs vs. anti-licensed PPIs) are of a similar syntactic, semantic or pragmatic nature.

In addition to theoretical considerations and behavioral judgments, physiological measures can be used to test whether and how NPIs and PPIs are treated differently by cortical processes during the course of sentence comprehension. Specifically, several studies have applied event-related potentials (ERPs) in order to specify qualitative aspects as well as the time-course of polarity processing (e.g., Saddy et al., 2004; Xiang et al., 2009; Yurchenko et al., 2013; Giannakidou and Etxeberria, 2018). The ERPs reported in this context are the N400 and the P600 component.

The N400 component was first reported in Kutas and Hillyard (1980) for semantic anomalies (e.g., *He spread his warm bread with socks*) in comparison to semantically sound sentences (e.g., the same sentence ending with *butter*). As is usually acknowledged, an enhanced N400 occurs due to lower degrees of semantic expectancy. While it is debated at which level and to what extent the N400 is tied to semantic processing, it is found robustly in language processing that involves lexical access difficulty or semantic integration costs (e.g., Kutas and van Petten, 1994; Lau et al., 2008; Kutas and Federmeier, 2011; Nieuwland et al., 2018). The N400 can arise due to different manipulations (e.g., priming, frequency, violations) that can be related to notions of predictability, plausibility, and similarity (see Nieuwland et al., 2018). Therefore, we resort to a broader interpretation of the N400 as a “change in a probabilistic representation of meaning” (see Rabovsky et al., 2018) at the lexical semantic or compositional semantic levels, in interaction with discourse context (Nieuwland and Van Berkum, 2006) or world knowledge (Hagoort et al., 2004) [see also the N400 effect found for other context-dependent phenomena such as presuppositions (Masia et al., 2017; Domaneschi et al., 2018), and metaphors (e.g., Bambini et al., 2016)].

The P600 is a positive deflection peaking at around 600ms post-stimulus with a centro-parietal or frontal distribution. It can be elicited by grammatical errors (Brown and Hagoort, 1999), syntactic complexity (Friederici et al., 2002) or difficult discourse contexts (van Herten et al., 2005; Burkhardt, 2007). In the earlier neurolinguistic literature, a P600 effect has been found for syntactically anomalous sentences such as *Das Hemd wurde am ^∗^gebügelt* (‘The shirt was on ^∗^ironed.’) relative to syntactically well-formed control sentences (cf. Hagoort et al., 1993; Osterhout and Hagoort, 1999; Friederici, 2002). A P600 effect has also been found for agreement errors (Coulson et al., 1998; Gunter et al., 2000), as well as for syntactically well-formed but locally ambiguous or complex sentences (Osterhout and Holcomb, 1992; Kaan et al., 2000; Kaan and Swaab, 2003). Thus, it has been identified as an indicator of syntactic repair or reanalysis, or of syntactic integration difficulty. However, later studies also report on a P600 effect (the so-called ‘semantic P600’) for syntactically well-formed but semantically ill-formed sentences, for example, due to the thematic role violation of verbs (e.g., *For breakfast, the eggs would only eat toast and jam.*) (Kuperberg et al., 2003). Furthermore, a P600 effect has also been reported for syntactically and semantically well-formed but pragmatically costly sentences (the so-called ‘pragmatic P600’). Burkhardt (2007), for example, relates a P600 effect to integration costs of new information into the discourse (e.g., *pistol* in *Yesterday a Ph.D. student was found dead* (vs. *shot*) *downtown. The press reported that the pistol was probably from army stocks*). These studies hint that the P600 might not specifically index syntactic processing but rather might reflect general processes of linguistic integration (Kuperberg, 2007; Friederici, 2011) or the internal monitoring of processing (van Herten et al., 2005; Sassenhagen et al., 2014; cf. Brouwer et al., 2017 for a neurocomputational model of ‘semantic/pragmatic P600’). Thus, the properties of the N400 and P600 are understood to a certain extent, making them useful in the context of the current study.

To our knowledge, Saddy et al. (2004) and Yurchenko et al. (2013) are the only two ERP studies that directly compare the processing of NPIs vs. PPIs. Both studies compared NPI violations to correct NPI conditions and observed an N400 component. However, the reanalysis of the same data from Saddy et al. (2004) showed a biphasic N400/P600 pattern for incorrect vs. correct NPI conditions (Drenhaus et al., 2004, 2006). The findings for PPIs are inconsistent. For PPI violations in comparison to correct PPIs, Saddy et al. (2004) report a biphasic N400/P600 pattern, but Yurchenko et al. (2013) report a P600-only effect. At present, the methodological sources of such different findings are unclear. They could be due to the different languages at study (i.e., German vs. Dutch), the different experimental designs or the different kinds of stimuli (see Table 1). Specifically, Saddy et al. (2004) used negative quantifiers (*e.g., kein Mann* ‘no man’) vs. indefinite expressions (e.g., *ein Mann* ‘a man’) plus a relative clause before the critical words. In contrast, Yurchenko et al. (2013) used negative adverbs (e.g., *niet* ‘not’) vs. different types of positive adverbs (e.g., *ook* ‘also’) immediately preceding the critical words. This means that the critical comparison in both studies compares items across negative vs. affirmative contexts. These contexts, however, are established at the beginning of the sentence in the Saddy et al.’s (2004) study versus next to the critical item in the Yurchenko et al.’s (2013) study. Furthermore, Yurchenko et al. (2013) used different words (e.g., *ook* ‘also’/*echt* ‘really’/*wel* ‘actually’) for creating affirmative contexts. The processing consequences of both design properties are unclear, but processing differences resulting from items appearing immediately before the critical item can also influence the waveform of the critical item (cf. Steinhauer and Drury, 2012). Concerning task and data evaluation, Saddy et al. (2004) collected behavioral data on the well-formedness of the test sentences in their study and they only used trials with correct answers for the ERP analysis. Yurchenko et al. (2013), in contrast, did not collect behavioral data but used comprehension questions for attention check and used all the collected trials for the ERP analysis. While it is debatable whether the physiological data recorded during sentence processing should be analyzed independent of separately recorded behavioral judgments or not, the decision may have an influence on the results. Here, we take the stance that behavioral data provide important information for the interpretation of the results, but that the discarding of ERP data based on behavioral results bears the danger of leaving aside an important aspect of processing for some linguistic phenomena. Another important difference between the two studies concerns the interpretation of the evoked potentials. Saddy et al. (2004) relate the P600 found for PPI violations vs. correct PPI conditions to syntactic integration costs based on earlier ERP literature (e.g., Osterhout and Holcomb, 1992; Friederici, 1995; Kaan et al., 2000; Friederici et al., 2002). In contrast, Yurchenko et al. (2013) interpret the P600 (also found in PPI violations compared to correct PPI conditions) pragmatically, namely, to discourse integration costs (cf. Burkhardt, 2007). Lastly, their conclusions are based on comparisons between violation conditions vs. correct conditions within the same polarity profile. This leaves open the possibility that the negative vs. affirmative contexts, which are necessarily different across NPIs and PPIs, influenced the results. This is possible as it is specifically known that negation creates neurophysiological processing costs in contrast to affirmation (cf. Bahlmann et al., 2011). Thus, it is still unclear how exactly the neurophysiological signatures of NPI vs. PPI processing compare to each other.

**Table 1:** Design of Saddy et al. (2004), Yurchenko et al. (2013), and the current study.

	Factors		Item example
		
	Context	Polarity profile	
Saddy et al., 2004; Drenhaus et al., 2006	Affirmative/negative	NPI/PPI	(i) (a) *Kein/^∗^Ein Mann, der einen Bart hatte, war jemals__NPI_ froh.* no/a man, who a beard had, was ever happy (b) *Ein/^∗^Kein Mann, der einen Bart hatte, war durchaus__PPI_ froh.* a/^∗^no man, who a beard had, was certainly happy
Yurchenko et al., 2013	Affirmative/negative	NPI/PPI/non-PI	(ii) (a).*..zijn handschrift was niet/^∗^ook bijster__NPI_ leesbaar.* his handwriting was not/^∗^also at all readable (b)...*partijen zijn echt/^∗^niet nogal__PPI_ ensgezind.* party the really/^∗^not rather unanimous (c)...*het was niet/^∗^wel bijzonder__nonPI_ chic.* it was not/actually particularly chic
Current study	Affirmative/negative	NPI/PPI/non-PI	cf. (3)


In summary, there is neither a consensus on the nature of NPIs and PPIs in theoretical linguistics, nor on their processing in the psycholinguistic literature (see also Shao and Neville, 1998; Xiang et al., 2009, 2016). The present study aims at resolving this inconsistency using a combination of behavioral and ERP measurements while investigating NPIs and PPIs in a balanced design. Previous studies focused on context violations, i.e., comparing the processing of the same polarity item in different contexts (Saddy et al., 2004; Yurchenko et al., 2013). Yet, as described above, those contexts are very different across the two studies and, in the case of Yurchenko et al. (2013) also across the different polarity conditions. Thus, it is not possible to draw more general conclusions about the processing of NPIs and PPIs *per se*. In order to address this problem, we chose to apply a strict control of linguistic context: NPIs, PPIs, and non-PIs were embedded in the same negative or positive sentence context. We collected behavioral and electrophysiological data and included all the trials in the ERP analysis, as we are interested in brain responses to all the stimuli, independent of the judgments. Moreover, we analyzed not only NPI and PPI violations relative to their respective correct sentences as previous studies, but also non-PIs in negative vs. affirmative contexts. The latter comparison is important as it enabled us to exclude the possibility that the effects found for NPIs and PPIs are due to having a negative vs. positive context. Crucially, for the first time to our knowledge, we also compared the difference between the violation condition vs. the correct condition of NPIs and that of PPIs. With the last comparison, we are able to draw more conclusive evidence about the underlying processes supporting the comprehension of NPIs vs. PPIs.

Using the violation paradigm, we tested German polarity items using a 2 × 3 factorial design, with the factors of ‘context’ (affirmative/negative) and ‘polarity profile’ (NPI/PPI/non-PI), see Table 1. The 6 conditions are exemplified in (3a-f). All sentences consisted of a main clause containing an affirmative (*den* ‘the’) or negative (*kein* ‘no’) noun phrase and an item of different polarity profile, namely, either the NPI *jemals* ‘ever,’ the PPI *schon* ‘already’ or the non-PI *sehr* ‘very’. A relative clause modifying the noun phrase is included in between to assure a clean baseline. In addition, we added two filler conditions involving semantic/pragmatic or morpho-syntactic violations (3g-h) to balance out the total numbers of incorrect and correct sentences.

(3) (a) *Peter hat **den Kuchen**, der viele Nüsse*        **(NPI-aff)**
  enthielt, **jemals** oft gebacken.
  Peter has the cake, which many nuts contains, ever often baked.
  ‘Peter has baked the cake ever often, which contains many nuts.’
  (b) *Peter hat **keinen***                                                 **(NPI-neg)**
  ***Kuchen***,..................................***jemals** oft gebacken.*
  Peter has no cake, which many nuts contains, ever often baked.
  ‘Peter has baked no cake ever often, which contains many nuts.’
  (c) *Peter hat **den***                                                         **(PPI-aff)**
  ***Kuchen***,.......................................***schon** oft gebacken.*
  Peter has the cake, which many nuts contains, already often baked.
  ‘Peter has baked the cake already often, which contains many nuts.’
  (d) *Peter hat **keinen***                                                  **(PPI-neg)**
  ***Kuchen**,.................................**schon** oft gebacken.*
  Peter has no cake, which many nuts contains, already often baked.
  ‘Peter has baked no cake already often, which contains many nuts.’
  (e) *Peter hat **den***                                                        **(non-PI-aff)**
  ***Kuchen**,......................................**sehr** oft gebacken.*
  Peter has the cake, which many nuts contains, very often baked.
  ‘Peter has baked the cake very often, which contains many nuts.’
  (f) *Peter hat **keinen***                                                  **(non-PI-neg)**
  ***Kuchen**,..................................**sehr** oft gebacken.*
  Peter has no cake, which many nuts contains, very often baked.
  ‘Peter has baked no cake very often, which contains many nuts.’
  (g) *Peter hat **keinen***                                                  **(anomaly_1)**
  ***Kuchen**,...............................sehr oft **gelernt**.?*
  Peter has no cake, which many nuts contains, ever often learned.
  ‘Peter has learned no cake very often, which contains many nuts.’
  h. *Peter hat **den***                                                          **(anomaly_2)**
  ***Kuchen**,......................................sehr oft **backen***.
  Peter has the cake, which many nuts contains, ever often bake.
  ‘Peter has bake the cake very often, which contains many nuts.’

Subjects were asked to read the sentences and rate their naturalness during the EEG measurements. Our main hypotheses based on the recent literature were twofold:

According to the assumption that NPI violations lead to ungrammaticality but PPI violations lead to pragmatic oddity (Liu, 2017; Iordachioaia and Liu, 2018), NPI and PPI violations would reduce the acceptability of sentences to different degrees. This leads to the following specific predictions with respect to the behavioral data: (1) There would be an interaction between the factors ‘context’ and ‘polarity profile’ in such a way that NPIs and PPIs require different supporting contexts. (2) NPI-aff and PPI-neg (i.e., NPIs and PPIs in non-supporting contexts) would be rated as significantly less natural than the other correct conditions; (3) NPI-aff (i.e., NPIs in non-supporting contexts) would be rated much less natural than PPI-neg (i.e., PPIs in non-supporting contexts).

In line with the theoretical accounts described above, NPI and PPI violations involve distinct physiological processes that lead to different signatures in the ERP analysis. Specifically, NPI violations are due to syntactic and semantic reasons (Klima, 1964; Ladusaw, 1979; Giannakidou, 1998; Chierchia, 2004), whereas PPI violations are due to semantic and pragmatic reasons (see Giannakidou, 2012; Liu and Iordachioaia, 2018). We thus derived the following predictions relating to the ERP measurements: (1) NPI-aff would elicit both N400 and P600 components in comparison to NPI-neg. (2) PPI-neg would elicit both N400 and P600 components in comparison to PPI-aff. (3) There would be no differences in N400 or P600 amplitude in the comparison of nonPI-neg vs. nonPI-aff. (4) Due to the different degree or nature of NPI vs. PPI violations, there would be differences in N400 or P600 amplitude in the comparison of the difference wave of NPI-aff vs. NPI-neg and that of PPI-neg vs. PPI-aff.

## Materials and Methods

### Participants

Native German-speaking students (*N* = 27) from Osnabrück University participated in the study. All procedures were approved by the local ethics committee and participants signed informed consent forms. Data of three subjects were excluded due to technical issues or excessive artifacts. Data of 24 subjects (mean age of 21.8 years, *SD* = 2.65, 12 female) were included in the final data analysis. Among them, 20 subjects were right-handed and 4 were left-handed. As left-handedness could go along with certain variations in topographies (in case the dominant hemispheres are reversed in some of the left-handers), this could create a potential confound. Please note that in the following, different conditions are compared only within subjects. We do not compare left-handed subjects vs. right-handed subjects at any point. Nevertheless, we do not make any claims about lateralized effects in the topographic results. All subjects had normal or corrected-to-normal vision, and had no history of neurological disorder or dyslexia. They received either payment or course credits for their participation.

### Materials

We used 120 sets of sentences in eight conditions, as exemplified in (3) above. This resulted in a total of 960 sentences (see the Supplementary Material), which we divided into three lists of equal length. The affirmative and corresponding negative versions, e.g., conditions (3a) and (3b), of a sentence item were always part of the same list. However, the 8 conditions based on an item were distributed across the lists, so that each item was repeated either 2 or 4 times in each list. This procedure guaranteed a sufficient number of test items for each subject, while limiting reuse of items for different conditions read by a subject. Thus, each list contained 320 sentences with 40 sentences per condition. Out of the three lists, we created six different final lists of identical length through pseudo-randomization of the sequence. The pseudo-randomization was done with the following constraints: First, for each list, there were at least 25 sentences between two sentences based on the same set. Second, the same condition never repeated three times in a row. Third, same patterns of transition in which one condition would always follow the other one were avoided. Each subject read one list of sentences.

### Procedure and Data Acquisition

We combined recording the physiological signature of sentence processing with a naturalness rating study. After the EEG set-up, subjects moved to a quiet room and sat in front of a computer screen at a distance of about 80 cm. The subjects were instructed to read each sentence word by word and answer the question of whether it sounds natural. The experiment started with a practice phase of eight sentences. Then, a list of 320 test sentences was presented word by word. Each word was shown in the center of a screen for 600 ms without pauses in between. A fixation cross was shown in the beginning of each sentence for 300 ms and after the final word again for 900 ms. Then, a happy and a sad emoticon were shown on the left and right position: half of the participants saw the happy emoticon on the left and the other half on the right. Subjects had to judge the naturalness of the sentence without time limitation by clicking on the respective emoticon on the screen. Once a response was given, the screen went blank for 500 ms before the next sentence began. The whole experiment took around an hour and was divided into four blocks of 80 sentences per block, i.e., subjects could take three breaks in between if they wanted.

For the EEG measurement, we used a 64-channel amplifier (BrainAmp DC, manufactured by *Brain Products, Germany*) with 62 equidistantly spaced Ag/AgCI electrodes fixed in an elastic cap (actiCAP, manufactured by *Brain Products, Germany*). Among these, the reference and ground electrodes were placed on the scalp with the Reference in the Cz position and the Ground in a more frontal location on the midline. Two additional EOG (electro-oculographic) electrodes were placed below the eyes on the infra-orbital ridge to monitor blinks, but subjects were told in advance not to move or blink during the measurement. With the software BrainVision Recorder (*Brain Products, Germany*), data were continuously recorded with a sampling rate of 500 Hz. All electrode impedances were kept below 5 kΩ.

### Data Analysis

The behavioral data were analyzed using a 2 × 3 factorial repeated measures ANOVA (analysis of variance).

All the ERP-data were pre-processed via the MATLAB toolbox EEGLAB (Delorme and Makeig, 2004). First, the data were re-referenced offline to the averaged left and right mastoids, with the previous reference electrode Cz added back, resulting in a total of 65 electrodes. Then, the relevant epochs for the target word, ranging from 200 ms before to 1500 ms after the stimulus onset, were cut out. This resulted in epochs of 1700 ms with the first 200 ms serving as the baseline. We chose long epochs to keep the analysis between this study and different studies that we are currently planning identical (e.g., with L2 learners and children). After that, an automatic artifact rejection via the EEGlab plugin FASTER (Nolan et al., 2010) was conducted, including band-pass filtering between 0.5Hz^4^ and 95Hz. Additional rejection of artifacts was done manually upon careful inspection. As a result, the total of 5760 trials from all the datasets were reduced by 10% to 5184 trials. The number of remaining trials was, however, almost the same across the conditions, ranging between 860 and 870 trials per condition. After the data preprocessing, the grand mean was calculated by averaging the single subject averages for each condition respectively.

We chose non-parametric statistical testing using state of the art cluster-based permutation tests implemented in the MATLAB toolbox FieldTrip (Oostenveld et al., 2010). Specifically, an ERP analysis compares different experimental conditions at different electrodes and different points in time. A naive approach of testing the ERP at each electrode and each point in time individually leads to a huge number of tests (#electrodes ^∗^ #time points) of noisy data (single measurements). Without multiple comparison correction this leads to many false positives (type-1 errors); with corrections for multiple comparisons this leads to an inflation of false negatives (type-2 errors). Cluster based tests accumulate evidence for a difference between conditions in the neighborhood of electrodes and time-points. This results in the sequence of first level *t*-tests, summing up evidence of neighboring electrodes and time points, and finally the appropriate statistical test. As a result, the cluster based permutation test evaluates the statistical significance of a cluster in space and time and thereby simultaneously reduces the type-1 and type-2 error rates. These advantages lead to the increasing use of this modern statistical approach (see e.g., Ehinger et al., 2015; Pernet et al., 2015 for a recent review on the statistical properties of such approaches). Additionally, the cluster based permutation test does not require assumptions about the specific topography of the tested effect. Instead, the set of electrodes with significant differences between conditions is returned as a result. Thus, instead of heuristics as to which topography to investigate, we obtain a data driven analysis of the relevant topography of the significant difference between conditions.

In more detail, first, a dependent-samples *t*-test was performed for each sample of the respective conditions, whereby *sample* refers to a (channel, time)-pair. Secondly, all samples with a *p*-value lower than the predefined significance threshold of 5%, were selected and pooled into clusters based on their temporal and spatial proximity. We specified 2 as the minimal number of neighborhood channels for a selected sample (i.e., a sample whose *t*-value exceeds the threshold) to be included in the clustering algorithm. Next, the *t*-values of all the samples from one cluster were summed up in order to provide a cluster-level statistics. The significance of the cluster-level statistics was estimated via a comparison with its randomization distribution that was obtained in the following way: all trials were put into a Monte-Carlo simulation with 1000 repetitions in order to calculate the cluster-level statistic. That is, all trials from the two respective conditions were collected in a single set from which a random partition was created. Then, the cluster-level statistic was calculated for the random partition just as described previously. These steps were repeated here for 1000 random partitions resulting in the randomization distribution of the test statistics. When comparing the latter to the actually observed cluster statistics, *p*-values were calculated based on the proportion of random partitions with a more significant test statistic than the actually observed ones. If the resulting *p*-value, that is, the probability of observing a more significant test statistic than the actually observed cluster-level statistic, was equal to or below the predetermined threshold, i.e., 5%, then the respective cluster was considered significant. This procedure follows the recommendations of Maris and Oostenveld (2007) closely.

In order to capture the full time range of N400 as well as P600 effects in question, we conducted the cluster-based permutation tests separately for two time windows: 350–500 ms post-stimulus (i.e., N400) and 500–800ms post-stimulus (i.e., P600). We selected this procedure for two reasons. First, based on earlier studies on N400 and P600 effects in general and related to the processing of polarity items, we expected to find effects for the two time windows. Thus, we focused the test on the respective temporal interval (see e.g., Wang et al., 2016 for using similar procedures). This enables us to interpret our results in comparison to the existing literature. Second, the permutation test is sensitive to the overall statistics within the time window considered. Therefore extending it greatly influences the control population (the permuted signal) and in the case of non-stationary statistics might negatively influence the statistical power. Thus, although the cluster permutation test is a data driven procedure, it can sometimes be narrowed down on relevant temporal regions, for instance, to avoid including other potentials (Song and Iverson, 2018). This said, the cluster permutation test still serves to detect the specific temporal and spatial extension of the effects.

We performed four tests on the following comparisons: NPI-aff vs. NPI-neg, PPI-neg vs. PPI-aff, nonPI-neg vs. nonPI-aff, and NPI-Diff (the difference of incorrect vs. correct NPIs) vs. PPI-Diff (the difference of incorrect vs. correct PPIs). For visualization, ERPs were filtered offline with a 10 Hz low pass filter but all statistical analyses were computed on non-filtered data.

## Results

### Behavioral Data

As a first step we investigated whether our participants judged the sentences according to the hypotheses presented in “Introduction.” Table 2 presents the mean ratings for all the eight conditions.

**Table 2:** Mean ratings of naturalness (0 = unnatural, 1 = natural) and SDs.

Conditions	Mean	*SD*
NPI-aff	0.20	0.15
NPI-neg	0.79	0.20
PPI-aff	0.94	0.07
PPI-neg	0.63	0.28
nonPI-aff	0.95	0.07
nonPI-neg	0.84	0.17
anomaly_1	0.08	0.20
anomaly_2	0.03	0.03


As Table 2 shows, the two anomaly control conditions (3g-h), whether due to semantically mismatching or unconjugated verbs, received extremely low ratings. This shows that subjects paid attention and understood the sentences. Ratings of sentences with a licensed NPI/PPI/non-PI were rated at 0.79 or above. In contrast, all sentences with unlicensed or anti-licensed use were rated 0.63 or below. In fact, separately considering PPI-neg, all other violations were rated very low at 0.20 (for NPI-aff) or below. Thus, the ratings by our participants clearly differentiated between licensed and unlicensed/anti-licensed usage.

A 2 × 3 ANOVA with ‘context’ (affirmative/negative) and ‘polarity profile’ (NPI/PPI/non-PI) as within-subjects factors was conducted on the six critical conditions. As our focus was on the processing of NPI violations, PPI violations and their differences, we conducted *post hoc* paired *t*-tests for four comparisons, with the results in Table 3.

**Table 3:** *t*-Tests by subjects and by items.

Comparisons	Conditions	*t*-Test results
1	NPI-aff vs. NPI-neg	*t*1(1,23) = -11.5, *p* < 0.001; *t*2(1,119) = -25.2, *p* < 0.001
2	PPI-neg vs. PPI-aff	*t*1(1,23) = -5.6, *p* < 0.001; *t*2(1,119) = -16.5, *p* < 0.001
3	nonPI-aff vs. nonPI-neg	*t*1(1,23) = 3.5, *p* = 0.002; *t*2(1,119) = 7.4, *p* < 0.001
4	NPI-aff vs. PPI-neg	*t*1(1,23) = -7.0, *p* < 0.001; *t*2(1,119) = -18.0, *p* < 0.001


According to the ANOVA, there was a main effect of ‘polarity profile’ by subjects and items [*F*1(2,22) = 123.6, *p* < 0.001, $\eta_{p}^{2}$ = 0.84; *F*2(2,118) = 370.2, *p* < 0.001, $\eta_{p}^{2}$ = 0.76], a main effect of ‘context’ only by items [*F*1(1,23) = 2.6, *p* = 0.123, $\eta_{p}^{2}$ = 0.10; *F*2(1,119) = 31.4, *p* < 0.001, $\eta_{p}^{2}$ = 0.21] and an interaction by subjects and items [*F*1(2,22) = 146.2, *p* < 0.001, $\eta_{p}^{2}$ = 0.86; *F*2(2,118) = 494.6, *p* < 0.001, $\eta_{p}^{2}$ = 0.81]. Specifically, non-PIs were rated more natural than PPIs, which were rated more natural than NPIs. Negative contexts were rated more natural than affirmative ones. More crucially, we see in the interaction that NPIs and PPIs require different supporting contexts. In the *post hoc* paired *t*-tests, all the four comparisons were highly significant by both subjects and items. Crucially, NPI violations were rated significantly worse than PPI violations, see Comparison 4 in Table 3. These data support all three predictions on the behavioral data put forward in Section “Introduction.”

### ERP Data

**FIGURE 1:**
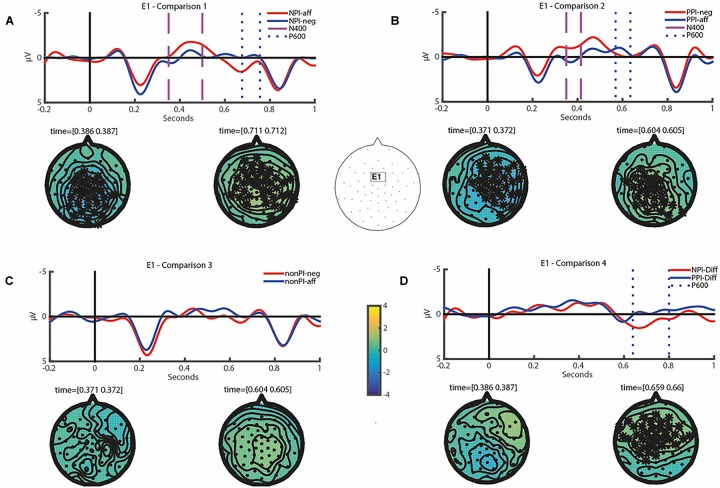
**(A–D)** Show grand averaged ERPs time-locked to the onset of the critical item including a baseline of 200 ms at the representative electrode E1 (see its scalp location at the center) for Comparison 1–4 as specified in Table 4. Two vertical lines of the same pattern and color are used to indicate the exact time window of a significant effect. In each of these comparisons, topographic isovoltage difference maps for the N400 and P600 time window are shown at the time point where the cluster had its maximum extension, with electrodes belonging to significant clusters highlighted by asterisks. For non-significant differences, we chose a time point for topographic maps that was significant in a different comparison where one of the conditions was involved.

As a next step we test the four predictions developed in Section “Introduction” relating to the ERP measurements:

After standard preprocessing of the EEG recordings, we performed a cluster-based permutation test as described in Section “Data Analysis.” It revealed a significant difference for NPI-aff relative to NPI-neg in both time windows of 350–500 ms (N400) and 500–800 ms (P600) post-stimulus (see Comparison 1 in Table 4 and Figure 1A). The N400 effect significant over the time window of [350 500] had a central distribution. The amplitude difference was -1.57 μV (*SD* = 2.91) at the selected representative electrode E1 (see Figure 1 for the electrode location) and at 386 ms where the effect is at its maximum. In the following, the amplitude difference in microvolt for a significant effect is always given for E1 at a time point with maximum effect. Note that the statistical test takes into account all electrodes and time points. However, to characterize the effect size we report the maximum value. This value is not individually tested, but states the effect size of an already tested and statistically significant effect of a whole cluster. The P600 effect significant for the time window of [677 756] had a central-parietal distribution with an amplitude difference of 1.49 μV (*SD* = 3.17) at 711ms. The results of this comparison confirm the first prediction that NPI-aff elicit both N400 and P600 components in comparison to NPI-neg.

**Table 4:** Statistical results of the cluster-based permutation test.

Comparisons	Conditions	Time windows	
		
		[350 500]	[500 800]
1	NPI-aff vs. NPI-neg	NC (^∗∗^), *t* = [350 500]	PC (^∗^), *t* = [677 756]
2	PPI-neg vs. PPI-aff	NC (^∗^), *t* = [350 415]	PC (^∗^), *t* = [569 635]
3	nonPI-aff vs. nonPI-neg	–	–
4	NPI-Diff vs. PPI-Diff	–	PC (^∗∗^), *t* = [639 800]


NC stands for a negative cluster; PC stands for a positive cluster. A significant difference with *p* < 0.05 is indicated with one asterisk ^∗^; a highly significant difference with *p* < 0.01 is indicated with two asterisks ^∗∗^; ‘–’ indicates the lack of a significant difference with *p* ≥ 0.05. The range for *t* indicates the time window of a significant effect.

Next, we tested the second prediction. The cluster-based permutation test revealed a significant difference for PPI-neg relative to PPI-aff across two time windows (see Comparison 2 in Table 4 and Figure 1B). Both the N400 (for the time window of [350 415]) and the P600 effect (for a relatively small time window of [569 635]) had a central-parietal distribution, with an amplitude difference of -1.87 μV (*SD* = 3.28) at 371 ms and 0.98 μV (*SD* = 2.22) at 604 ms respectively. Thus, our results support the second prediction that PPI-neg would elicit both N400 and P600 components in comparison to PPI-aff.

The next comparison concerning non-PIs in negative vs. affirmative conditions addresses the third prediction. In fact, it did not reveal an N400 or P600 effect, see Comparison 3 in Table 4 and Figure 1C. These data support the third hypothesis that there would be no differences in N400 or P600 amplitude in the comparison of nonPI-neg vs. nonPI-aff. This indicates that non-PIs are processed differently from NPIs or PPIs. Furthermore, this also shows that just the manipulation of context by itself did not influence the results in the other comparisons.

The results of the three comparisons altogether suggest that NPI and PPI violations might involve similar processes. This, however, was dis-confirmed in the comparison between the difference wave of NPI violations (i.e., NPI-Diff: NPI-aff vs. NPI-neg) vs. that of PPI violations (i.e., PPI-Diff: PPI-neg vs. PPI-aff). Here, while we did not find an N400 effect, we found a P600 effect significant for the time window of [639 800] for NPI-Diff relative to PPI-Diff (see Comparison 4 in Table 4 and Figure 1D) with an amplitude difference of 2.51 μV (*SD* = 3.28) at 659 ms where the effect is at its maximum.

As we can see from Table 4 and Figure 1, NPI-aff elicited both N400 and P600 effects, in comparison to NPI-neg. PPI-neg elicited both N400 and P600 effects in comparison to PPI-aff. NPI-Diff, in comparison to PPI-Diff, elicited a significant P600 effect with no N400 effect. This supports the fourth hypothesis that there would be differences in N400 or P600 amplitude in the comparison of NPI-Diff vs. PPI-Diff.

## Discussion

In the current study, we tested German NPIs, PPIs and non-PIs in a full factorial design to find out whether NPIs and PPIs are processed similarly. The results in the behavioral responses show, as was predicted, that sentences with NPI or PPI violations both received lower naturalness ratings than correct NPI/PPI/non-PI sentences and that NPI violations were judged significantly less natural than PPI violations. Independently of the behavioral data, the ERP data show the following results: (1) NPI violations (i.e., NPI-aff) elicited both N400 and P600 components in comparison to NPI-neg; (2) PPI violations (i.e., PPI-neg) elicited both N400 and P600 components in comparison to PPI-aff; (3) NPI violations (i.e., NPI-Diff) elicited a P600 effect relative to PPI violations (i.e., PPI-Diff). These findings taken together speak for the main hypothesis that NPIs and PPIs involve distinct processes.

The behavioral data show that non-PIs are well-formed in both affirmative and negative contexts. In contrast, NPIs are well-formed in negative contexts but considered unnatural in affirmative contexts. PPIs are well-formed in affirmative contexts but considered somewhat unnatural, yet, not completely unnatural in negative contexts. The relatively high rating for PPI-neg is compatible with the observation that PPIs following negation do not always result in ungrammaticality (Horn, 1989; Szabolcsi, 2004; Liu, 2017). The different ratings of NPI-aff and PPI-neg suggest that the two kinds of violations are not of the same degree. The behavioral data are by and large in line with the literature, thus, the congruence of behavioral results supports the validity and general relevance of the experimental design.

While behavioral data cannot pin down the nature of this difference, ERP measures can provide additional, independent evidence revealing their differences in the underlying processes. For the ERP analysis, we included all the trials without artifacts independently of the behavioral responses, whereas Saddy et al. (2004) only used trials with correct judgments (i.e., NPI-neg and PPI-aff as well-formed; NPI-aff and PPI-neg as ill-formed) and without artifacts in the judgment task (79.3% of all trials). On the surface, our results resemble those of Saddy et al. (2004) and Yurchenko et al. (2013) in that they all speak for distinct processes between NPIs and PPIs, but the details are different. Furthermore, our ERP findings also differ from Drenhaus et al. (2006). They found a biphasic N400/P600 pattern for both NPI and PPI violations in comparison to their respective correct conditions, whereas we found a P600 effect for NPI vs. PPI violations. Most crucially, due to our additional comparison between NPI and PPI violations, we can more precisely pin down the processing similarities and differences between them, see Table 5 for the comparison of the ERP results. We will now discuss and interpret the results for the two time windows.

**Table 5:** Comparison of ERP results.

Paper	Language	Comparison		
		
		NPI-aff vs. NPI-neg	PPI-neg vs. PPI-aff	NPI-Diff vs. PPI-Diff
Current study	German	N400+P600	N400+P600	P600
Saddy et al., 2004	German	N400	N400+P600	–
Drenhaus et al., 2006	German	N400+P600	N400+P600	–
Yurchenko et al., 2013	Dutch	N400	P600	–


### 350–500 ms Time Window (N400)

Concerning NPIs, Saddy et al. (2004), the first ERP study on German polarity items, found an N400 effect in the comparison of unlicensed vs. licensed NPIs, which they relate to semantic integration costs. Schütte (2006) conducted an ERP study on NPIs with the context varying between negation and *wh*-questions (i.e., a weaker NPI licensor than negation, c.f. van der Wouden, 1997) and found that the latter evoked a larger N400 than negation. In their study on Dutch polarity items, Yurchenko et al. (2013) also found an N400 effect in the comparison of unlicensed vs. licensed NPIs (even though the impact of the different words immediately before the critical items on the N400 is unclear). In our study, we found an N400 effect with a centro-parietal distribution for NPI-aff in comparison to NPI-neg, which replicates the findings of the mentioned papers (cf. Steinhauer et al., 2010 that did not find an N400 effect).

Concerning PPIs, Saddy et al. (2004) found an N400 effect for PPIs in negation in comparison to PPIs in affirmation, whereas Yurchenko et al. (2013) did not. In our study, we found an N400 effect with a centro-parietal distribution in the comparison of PPI-neg vs. PPI-aff. Despite the differences in the design and in the data analysis, our result is similar to that of Saddy et al. (2004), providing convergent evidence that anti-licensed PPIs increase semantic processing costs.

In addition, we also compared NPI and PPI violations directly, which has not been done in previous literature. We did not find any significant N400 effect, that is, there was no graded N400 effect. By this, we conclude that both violations increase semantic processing costs in a similar way (or to a similar extent).

### 500–800 ms Time Window (P600)

Concerning NPIs, neither Saddy et al. (2004) nor Yurchenko et al. (2013) found a P600 effect for unlicensed vs. licensed NPIs. In a follow-up study, Drenhaus et al. (2004) found the N400 as well as a P600 for unlicensed NPIs, contradicting the results of Saddy et al. (2004). Due to the inconsistency, Drenhaus et al. (2006) conducted a *post hoc* analysis on the data from the two previous studies using an advanced ERP analysis method – symbolic resonance analysis (SNR). This analysis revealed a P600 effect for unlicensed NPIs, which had not been found by Saddy et al. (2004) via common averaging techniques. Drenhaus et al. (2006) therefore conclude that NPI violations do not only increase semantic processing costs but under their assumption about the functional role of P600, also syntactic processing costs. Yurchenko et al. (2013) argue that the presence of P600 in previous studies (Shao and Neville, 1998; Saddy et al., 2004, as reanalyzed in Drenhaus et al., 2006), in contrast to the lack of it in their own study, is due to the degree of structural complexity in the stimuli. Saddy et al. (2004), for example, used an intervening relative clause between the licensor and the NPI, whereas in Yurchenko et al. (2013) the NPI immediately follows the licensor, i.e., sentence negation. It is a shortcoming of the design, as the lexical access to negation and its integration into the context might influence the processing of the NPI in working memory. In our study, the critical words were separated from the licensing or anti-licensing context by a relative clause as in Saddy et al.’s (2004) study. We did so to avoid unwanted effects of immediately preceding negation and to guarantee a clean baseline. We found a P600 effect for unlicensed NPIs that was robust in comparison to licensed NPIs, which is compatible with the finding of Drenhaus et al. (2006), despite the differences in data analyses.

Concerning PPIs, both Saddy et al. (2004) and Yurchenko et al. (2013) found a P600 effect for PPI violations in comparison to licensed PPIs. Saddy et al. (2004) relate the P600 effect to attempts of syntactic repair or reanalysis to interpret the PPI *durchaus* ‘certainly’ out of the scope of negation. This possibility is not likely to hold for our study, as the PPI *schon* ‘already’ does not only reject higher negation but also lower negation in German. Thus, the sentence *Peter hat schon keinen Kuchen gebacken*. (‘Peter has already baked no cake.’) is also ill-formed (Löbner, 1999). Yurchenko et al. (2013) propose an alternative account that the P600 elicited by unlicensed PPIs might be due to a search for a licensor in the wider discourse context. However, in their study PPIs immediately followed negation, whose processing might influence the waveform of the PPIs. Due to the specific structure of the stimuli, Yurchenko et al.’s (2013) study has limited implications for the contextual requirements of PPIs in general. In our study, using a clean baseline, we also found a P600 effect for anti-licensed PPIs vs. licensed PPIs (i.e., PPI-neg vs. PPI-aff), though the effect was for a short time window.

Due to the non-homogeneity of P600 effects, it is not clear whether the P600 effect from PPI violations is the same as that from NPI violation. It is also not clear whether the P600 found for PPI violations in all of the three studies has the same source. To clarify whether the P600 for NPIs and PPIs are distinct, the comparison (see Table 4, Comparison 4; Figure 1D) we performed between the difference waveform for incorrect vs. correct NPIs and that for incorrect vs. correct PPIs was revealing. While both violations elicited N400 effects of similar amplitude, the NPI-Diff triggered an increase in P600 amplitude in comparison to PPI-Diff. From this, we can conclude that the processing costs involved in the NPI and PPI violations are not the same.

To make sense of these results, we need to reconsider the controversy in the NPI/PPI research. Following the works of Klima (1964); Ladusaw (1979), and Giannakidou (1998), researchers mostly agree that sentences with unlicensed NPIs are ungrammatical for both structural and semantic reasons. In contrast, the nature of PPIs and their violation is currently highly debated (e.g., Szabolcsi, 2004; Homer, 2011; Nicolae, 2017; Zeijstra, 2017; Hoeksema, 2018). One important reason for the debate is that PPIs are shown to be rescuable as in (2). For example, our test sentences with the PPI *schon* in negation can be made natural through supporting discourse context, as in (4).

(4) A: *Peter hat den Kuchen* ***schon*** *oft gebacken, glaube ich*.
  (Peter has baked the cake already often, I think.)
  B: *Peter? Er hat* ***keinen*** *Kuchen* ***schon*** *oft gebacken! Der kann gar nicht backen*.
  (Peter? He has baked no cake already often! He cannot bake at all.)

This kind of rescuing strategy does not seem to be available for NPIs such as *jemals.* For such reasons, PPIs in negation do not make a sentence necessarily ungrammatical, but rather pragmatically odd. This view is compatible with the results of the behavioral data where PPI violations received significantly higher ratings than NPI violations. Furthermore, regarding the ERP components, Coulson et al. (1998) claim that the more salient a violation becomes, the bigger the P600 effect gets. In our study, the differences in the behavioral and ERP data might be due to a difference in salience between NPI and PPI violations. Syntactic violations resulting in ungrammaticality in the former case might be more salient than reparable pragmatic oddities in the latter case.

There is, however, another alternative explanation for this P600 effect. To compare and tease apart different P600s, Regel et al. (2014) report on an ERP study on morpho-syntactic and pragmatic manipulations. They found a P600 for morpho-syntactic violations in comparison to morpho-syntactically well-formed sentences and also for non-literal (i.e., ironic) sentences in comparison to literal sentences. However, in the direct comparison of the syntax-related P600 with the irony-related P600, they found differences in scalp distribution. Based on these, they conclude that morpho-syntactic violations and non-literal sentences involve distinct neurocognitive processes, namely, reanalysis of the sentence structure vs. pragmatic reanalysis. Related to polarity items, Xiang et al. (2016) compare unlicensed NPIs (e.g., 5a) as well as licensed NPIs by implicit negation of emotive expressions (e.g., *be surprised*, in 5c) respectively with licensed NPIs by explicit negation of negative quantifiers (e.g., *no* in 5b). They report a P600 effect for unlicensed NPIs vs. licensed NPIs with explicit negation, and an effect with a similar amplitude for licensed NPIs with implicit negation vs. licensed NPIs with explicit negation. The two effects have similar topographic distributions albeit at slightly different time windows. They attempt to distinguish the two P600 effects by different sources. More specifically, they relate the P600 effect for the unlicensed NPI condition vs. the licensed NPI condition with explicit negation to the “failure to construct a well-formed grammatical representation.” In comparison, they relate the P600 effect for the licensed NPI condition with emotive expressions vs. with explicit negation to pragmatic processing costs that arise in integrating an NPI into the context with an implicit negation.

(5) (a) *The dogs Andrew owns have ever responded to commands*.
  (b) *No dogs Andrew owns have ever responded to commands*.
  (c) *Andrew is surprised that the dogs he owns have ever responded to commands*.

Following the interpretations of the P600 in these two studies, it seems plausible that the P600 amplitude difference between NPI and PPI violations might reflect a difference between syntactic reanalysis (‘syntactic P600’) in the former case and pragmatic reanalysis (‘pragmatic P600’) in the latter. While the functional role of P600 is under constant debate, it is assumed to index processing costs on a more global level. In our study, we take it to have resulted from syntactic repair for NPIs. In the case of PPIs, we take it to have resulted from discourse (i.e., broad context) updating strategies, for example, to derive the speaker’s meaning as exemplified in (4) (see also Burkhardt (2007); Spotorno et al. (2013), and Bambini et al. (2016) for the pragmatic P600).

### Summary

Taking the behavioral and the ERP results together, we see a difference between NPI vs. PPI violations in the behavioral data, and a larger effect on the P600 in the ERP results for NPI relative to PPI violations. Prior to this study, it was not possible to draw conclusions concerning processing differences between NPIs and PPIs. Our study includes a control condition for affirmative vs. negative contexts and a direct comparison of NPI and PPI-related ERPs. We showed that differences were not due to negative or affirmative context alone and that NPIs involve additional, presumably syntactic, costs compared to PPIs.

To sum up, we would like to briefly address the scope and limitation of the current study. In general, polarity items can differ from one another greatly despite their shared sensitivity toward negation or negation-like contexts (Giannakidou, 2012). Furthermore, there is considerable variation within NPIs and within PPIs respectively (Hoeksema, 2018). The results we obtained in our study are certainly contingent on, for example, the specific experimental design, the specific polarity items and the specific contexts used. Thus, whether and to what extent they apply to other polarity items needs to be investigated in further studies. With these factors taken into consideration, we used the frequent NPI/PPI/non-PI *jemals/schon/very* in the strictly controlled contexts to make the results of general relevance not only for polarity processing but also for language processing in general. The finding of differences between NPI and PPI violations does not only provide novel perspectives on the processing of polarity items but it also has implications for accounts of NPIs and PPIs in theoretical linguistics.

## Conclusion

To sum up, while both NPI and PPI violations involve processing costs, these costs arise for different reasons. The similar N400 effect indicates that NPI and PPI violations increase semantic processing costs in a similar way. As the first study to compare NPI and PPI violation effects directly, we found a difference in the P600 effect for NPI violations relative to PPI violations. Thus, our results speak for partly distinct processes for NPI and PPI violations. In addition, the results and the interpretations of our data have further implications regarding existing theoretical accounts of polarity items. The lack of an N400 effect in the comparison between NPI and PPI violations is in line with semantic theories of NPIs/PPIs (Ladusaw, 1979; Giannakidou, 1998, 2012; Chierchia, 2004), reflecting the semantic restrictions/requirements on linguistic context. The increase in P600 amplitude of NPI violations relative to PPI violations can be due to their different nature. The former are syntactic anomalies (Klima, 1964; Chierchia, 2004) triggering reanalysis of the sentence structure, whereas the latter are pragmatic oddities (e.g., Horn, 1989; Liu, 2017) that induce reanalysis of the discourse context.

## Ethics Statement

This study was carried out in accordance with the Declaration of Helsinki (seventh version, 2013). All subjects gave written informed consent. The protocol was approved by the ethics committee of Osnabrück University.

## Author Contributions

ML, PK, and JM conceived and planned the experiment. ML and JM carried out the experiment and conducted data analyses. All the three authors contributed to the interpretation of the results. ML took the lead in writing the manuscript, with contributions of PK and JM.

## Conflict of Interest Statement

The authors declare that the research was conducted in the absence of any commercial or financial relationships that could be construed as a potential conflict of interest.

## Footnotes

1 Definition: A function F is downward monotonic iff for x⊆y, F(y) ⇒ F(x). For example, the sentence negation *not* is a downward monotonic function: When it applies to x and y with x ⊆ (i.e., being a subset of, or entailed by) y, e.g., x = *Tom likes big cities* and y = *Tom likes cities, Tom doesn’t like cities* ⇒ (i.e., entails that) *Tom doesn’t like big cities*.

2 Definition: A function F is non-veridical iff F(x) =/⇒ x. By this definition, the sentence negation *not*, for example, is a nonveridical function: When it applies to x, e.g., x = *Tom likes big cities, Tom doesn’t like big cities* =/⇒ (i.e., doesn’t entail that) *Tom likes big cities*.

3 Definition. A function F is anti-additive iff F(x ∪ y) = F(x) ∩ F(y). By this definition, the sentence negation *not*, for example, is an anti-additive function: When it applies to x and y with x ∪ y (i.e., x or y), e.g., x = *Tom likes big cities*, and y = *Tom likes small towns*, then *Tom doen’t like big cities or small towns = Tom doesn’t likes big cities* ∩ (i.e., and) *Tom doesn’t like small towns.*

4 Kappenman and Luck (2010) point out the high-pass filter can influence the ERP (see **Figure 7** of the paper). Nevertheless, as we are interested in the comparison between different conditions, it is important that the data are processed similarly. Furthermore, a band limit of 0.5 Hz corresponds to a time constant of 2 s. Setting the high-pass to e.g., 0.1 Hz leads to a time constant of 10 s. This means that the signal might be influenced by events at large temporal distances, which we consider to be undesirable.
