# Supplementary material for: Novel ERP Evidence for Processing Differences Between Negative and Positive Polarity Items in German
Source: Front Psychol. 2019 Mar 6;10:376. doi: 10.3389/fpsyg.2019.00376 (PMC6414433; doi:10.3389/fpsyg.2019.00376)
Supplement: Supplementary file 1 [file Table_1.DOCX]

Supplementary Material

**Novel ERP Evidence for Processing Differences between Negative and Positive Polarity Items in German**

Mingya Liu*, Peter König, Jutta L. Mueller

*** Correspondence:** Mingya Liu: liu.mingya@uni-osnabrueck.de

# Supplementary Figures / test sentences

1

(1) Peter hat den Kuchen, der viele Nüsse enthielt, jemals oft gebacken.

(2) Peter hat keinen Kuchen, der viele Nüsse enthielt, jemals oft gebacken.

(3) Peter hat den Kuchen, der viele Nüsse enthielt, schon oft gebacken.

(4) Peter hat keinen Kuchen, der viele Nüsse enthielt, schon oft gebacken.

(5) Peter hat den Kuchen, der viele Nüsse enthielt, sehr oft gebacken.

(6) Peter hat keinen Kuchen, der viele Nüsse enthielt, sehr oft gebacken.

(7) Peter hat keinen Kuchen, der viele Nüsse enthielt, sehr oft gelehrt.

(8) Peter hat den Kuchen, der viele Nüsse enthielt, sehr oft backen.

2

(9) Kim hat den Mann, der eine Brille trug, jemals lange geliebt.

(10) Kim hat keinen Mann, der eine Brille trug, jemals lange geliebt.

(11) Kim hat den Mann, der eine Brille trug, schon lange geliebt.

(12) Kim hat keinen Mann, der eine Brille trug, schon lange geliebt.

(13) Kim hat den Mann, der eine Brille trug, sehr lange geliebt.

(14) Kim hat keinen Mann, der eine Brille trug, sehr lange geliebt.

(15) Kim hat keinen Mann, der eine Brille trug, sehr lange gebacken.

(16) Kim hat den Mann, der eine Brille trug, sehr lange lieben.

3

(17) Lisa hat den Freund, der sie enttäuscht hatte, jemals lange gehasst.

(18) Lisa hat keinen Freund, der sie enttäuscht hatte, jemals lange gehasst.

(19) Lisa hat den Freund, der sie enttäuscht hatte, schon lange gehasst.

(20) Lisa hat keinen Freund, der sie enttäuscht hatte, schon lange gehasst.

(21) Lisa hat den Freund, der sie enttäuscht hatte, sehr lange gehasst.

(22) Lisa hat keinen Freund, der sie enttäuscht hatte, sehr lange gehasst.

(23) Lisa hat keinen Freund, der sie enttäuscht hatte, sehr lange gelesen.

(24) Lisa hat den Freund, der sie enttäuscht hatte, sehr lange hassen.

4

(25) Tom hat den Sportler, der harte Drogen nahm, jemals lange bewundert.

(26) Tom hat keinen Sportler, der harte Drogen nahm, jemals lange bewundert.

(27) Tom hat den Sportler, der harte Drogen nahm, schon lange bewundert.

(28) Tom hat keinen Sportler, der harte Drogen nahm, schon lange bewundert.

(29) Tom hat den Sportler, der harte Drogen nahm, sehr lange bewundert.

(30) Tom hat keinen Sportler, der harte Drogen nahm, sehr lange bewundert.

(31) Tom hat keinen Sportler, der harte Drogen nahm, sehr lange gegessen.

(32) Tom hat den Sportler, der harte Drogen nahm, sehr lange bewundern.

5

(33) Leon hat den Schüler, der zu dick war, jemals lange geärgert.

(34) Leon hat keinen Schüler, der zu dick war, jemals lange geärgert.

(35) Leon hat den Schüler, der zu dick war, schon lange geärgert.

(36) Leon hat keinen Schüler, der zu dick war, schon lange geärgert.

(37) Leon hat den Schüler, der zu dick war, sehr lange geärgert.

(38) Leon hat keinen Schüler, der zu dick war, sehr lange geärgert.

(39) Leon hat keinen Schüler, der zu dick war, sehr lange gegossen.

(40) Leon hat den Schüler, der zu dick war, sehr lange ärgern.

6

(41) Julia hat die Kollegin, die schlechte Laune hatte, jemals oft getroffen.

(42) Julia hat keine Kollegin, die schlechte Laune hatte, jemals oft getroffen.

(43) Julia hat die Kollegin, die schlechte Laune hatte, schon oft getroffen.

(44) Julia hat keine Kollegin, die schlechte Laune hatte, schon oft getroffen.

(45) Julia hat die Kollegin, die schlechte Laune hatte, sehr oft getroffen.

(46) Julia hat keine Kollegin, die schlechte Laune hatte, sehr oft getroffen.

(47) Julia hat keine Kollegin, die schlechte Laune hatte, sehr oft gekocht.

(48) Julia hat die Kollegin, die schlechte Laune hatte, sehr oft treffen.

7

(49) Melanie hat das Kind, das sich verletzt hatte, jemals lange betreut.

(50) Melanie hat kein Kind, das sich verletzt hatte, jemals lange betreut.

(51) Melanie hat das Kind, das sich verletzt hatte, schon lange betreut.

(52) Melanie hat kein Kind, das sich verletzt hatte, schon lange betreut.

(53) Melanie hat das Kind, das sich verletzt hatte, sehr lange betreut.

(54) Melanie hat kein Kind, das sich verletzt hatte, sehr lange betreut.

(55) Melanie hat kein Kind, das sich verletzt hatte, sehr lange gekauft.

(56) Melanie hat das Kind, das sich verletzt hatte, sehr lange betreuen.

8

(57) Bettina hat den Kollegen, der viel Erfahrung hatte, jemals lange respektiert.

(58) Bettina hat keinen Kollegen, der viel Erfahrung hatte, jemals lange respektiert.

(59) Bettina hat den Kollegen, der viel Erfahrung hatte, schon lange respektiert.

(60) Bettina hat keinen Kollegen, der viel Erfahrung hatte, schon lange respektiert.

(61) Bettina hat den Kollegen, der viel Erfahrung hatte, sehr lange respektiert.

(62) Bettina hat keinen Kollegen, der viel Erfahrung hatte, sehr lange respektiert.

(63) Bettina hat keinen Kollegen, der viel Erfahrung hatte, sehr lange gesungen.

(64) Bettina hat keinen Kollegen, der viel Erfahrung hatte, sehr lange respektieren.

9

(65) Michael hat den Sänger, der auf Englisch sang, jemals lange bewundert.

(66) Michael hat keinen Sänger, der auf Englisch sang, jemals lange bewundert.

(67) Michael hat den Sänger, der auf Englisch sang, schon lange bewundert.

(68) Michael hat keinen Sänger, der auf Englisch sang, schon lange bewundert.

(69) Michael hat den Sänger, der auf Englisch sang, sehr lange bewundert.

(70) Michael hat keinen Sänger, der auf Englisch sang, sehr lange bewundert.

(71) Michael hat keinen Sänger, der auf Englisch sang, sehr lange gefahren.

(72) Michael hat den Sänger, der auf Englisch sang, sehr lange bewundern.

10

(73) Alexander hat das Kind, das viele Freunde hatte, jemals lange gemocht.

(74) Alexander hat kein Kind, das viele Freunde hatte, jemals lange gemocht.

(75) Alexander hat das Kind, das viele Freunde hatte, schon lange gemocht.

(76) Alexander hat kein Kind, das viele Freunde hatte, schon lange gemocht.

(77) Alexander hat das Kind, das viele Freunde hatte, sehr lange gemocht.

(78) Alexander hat kein Kind, das viele Freunde hatte, sehr lange gemocht.

(79) Alexander hat kein Kind, das viele Freunde hatte, sehr lange gespart.

(80) Alexander hat das Kind, das viele Freunde hatte, sehr lange mögen.

11

(81) Emma hat das Pferd, das eine Verletzung hatte, jemals oft geritten.

(82) Emma hat kein Pferd, das eine Verletzung hatte, jemals oft geritten.

(83) Emma hat das Pferd, das eine Verletzung hatte, schon oft geritten.

(84) Emma hat kein Pferd, das eine Verletzung hatte, schon oft geritten.

(85) Emma hat das Pferd, das eine Verletzung hatte, sehr oft geritten.

(86) Emma hat kein Pferd, das eine Verletzung hatte, sehr oft geritten.

(87) Emma hat kein Pferd, das eine Verletzung hatte, sehr oft renoviert.

(88) Emma hat das Pferd, das eine Verletzung hatte, sehr oft reiten.

12

(89) Lukas hat den Mitarbeiter, der im Erdgeschoss arbeitete, jemals oft gesehen.

(90) Lukas hat keinen Mitarbeiter, der im Erdgeschoss arbeitete, jemals oft gesehen.

(91) Lukas hat den Mitarbeiter, der im Erdgeschoss arbeitete, schon oft gesehen.

(92) Lukas hat keinen Mitarbeiter, der im Erdgeschoss arbeitete, schon oft gesehen.

(93) Lukas hat den Mitarbeiter, der im Erdgeschoss arbeitete, sehr oft gesehen.

(94) Lukas hat keinen Mitarbeiter, der im Erdgeschoss arbeitete, sehr oft gesehen.

(95) Lukas hat keinen Mitarbeiter, der im Erdgeschoss arbeitete, sehr oft getrunken.

(96) Lukas hat den Mitarbeiter, der im Erdgeschoss arbeitete, sehr oft sehen.

13

(97) Laura hat das Gericht, das rohen Fisch enthielt, jemals oft gegessen.

(98) Laura hat kein Gericht, das rohen Fisch enthielt, jemals oft gegessen.

(99) Laura hat das Gericht, das rohen Fisch enthielt, schon oft gegessen.

(100) Laura hat kein Gericht, das rohen Fisch enthielt, schon oft gegessen.

(101) Laura hat das Gericht, das rohen Fisch enthielt, sehr oft gegessen.

(102) Laura hat kein Gericht, das rohen Fisch enthielt, sehr oft gegessen.

(103) Laura hat kein Gericht, das rohen Fisch enthielt, sehr oft besucht.

(104) Laura hat das Gericht, das rohen Fisch enthielt, sehr oft essen.

14

(105) Vanessa hat die Pflanze, die ihrer Mitbewohnerin gehörte, jemals oft gegossen.

(106) Vanessa hat keine Pflanze, die ihrer Mitbewohnerin gehörte, jemals oft gegossen.

(107) Vanessa hat die Pflanze, die ihrer Mitbewohnerin gehörte, schon oft gegossen.

(108) Vanessa hat keine Pflanze, die ihrer Mitbewohnerin gehörte, schon oft gegossen.

(109) Vanessa hat die Pflanze, die ihrer Mitbewohnerin gehörte, sehr oft gegossen.

(110) Vanessa hat keine Pflanze, die ihrer Mitbewohnerin gehörte, sehr oft gegossen.

(111) Vanessa hat keine Pflanze, die ihrer Mitbewohnerin gehörte, sehr oft geritten.

(112) Vanessa hat die Pflanze, die ihrer Mitbewohnerin gehörte, sehr oft gießen.

15

(113) Wolfgang hat den Kurs, der viel Zeit kostete, jemals lange besucht.

(114) Wolfgang hat keinen Kurs, der viel Zeit kostete, jemals lange besucht.

(115) Wolfgang hat den Kurs, der viel Zeit kostete, schon lange besucht.

(116) Wolfgang hat keinen Kurs, der viel Zeit kostete, schon lange besucht.

(117) Wolfgang hat den Kurs, der viel Zeit kostete, sehr lange besucht.

(118) Wolfgang hat keinen Kurs, der viel Zeit kostete, sehr lange besucht.

(119) Wolfgang hat keinen Kurs, der viel Zeit kostete, sehr lange getroffen.

(120) Wolfgang hat den Kurs, der viel Zeit kostete, sehr lange besuchen.

16

(121) Lea hat die Suppe, die ihrem Bruder schmeckte, jemals oft gekocht.

(122) Lea hat keine Suppe, die ihrem Bruder schmeckte, jemals oft gekocht.

(123) Lea hat die Suppe, die ihrem Bruder schmeckte, schon oft gekocht.

(124) Lea hat keine Suppe, die ihrem Bruder schmeckte, schon oft gekocht.

(125) Lea hat die Suppe, die ihrem Bruder schmeckte, sehr oft gekocht.

(126) Lea hat keine Suppe, die ihrem Bruder schmeckte, sehr oft gekocht.

(127) Lea hat keine Suppe, die ihrem Bruder schmeckte, sehr oft gelesen.

(128) Lea hat die Suppe, die ihrem Bruder schmeckte, sehr oft kochen.

17

(129) Uwe hat die Zeitschrift, die schlechte Qualität hatte, jemals oft gekauft.

(130) Uwe hat keine Zeitschrift, die schlechte Qualität hatte, jemals oft gekauft.

(131) Uwe hat die Zeitschrift, die schlechte Qualität hatte, schon oft gekauft.

(132) Uwe hat keine Zeitschrift, die schlechte Qualität hatte, schon oft gekauft.

(133) Uwe hat die Zeitschrift, die schlechte Qualität hatte, sehr oft gekauft.

(134) Uwe hat keine Zeitschrift, die schlechte Qualität hatte, sehr oft gekauft.

(135) Uwe hat keine Zeitschrift, die schlechte Qualität hatte, sehr oft betreut.

(136) Uwe hat die Zeitschrift, die schlechte Qualität hatte, sehr oft kaufen.

18

(137) Hannah hat das Referat, das sie halten musste, jemals lange vorbereitet.

(138) Hannah hat kein Referat, das sie halten musste, jemals lange vorbereitet.

(139) Hannah hat das Referat, das sie halten musste, schon lange vorbereitet.

(140) Hannah hat kein Referat, das sie halten musste, schon lange vorbereitet.

(141) Hannah hat das Referat, das sie halten musste, sehr lange vorbereitet.

(142) Hannah hat kein Referat, das sie halten musste, sehr lange vorbereitet.

(143) Hannah hat kein Referat, das sie halten musste, sehr lange geritten.

(144) Hannah hat das Referat, das sie halten musste, sehr lange vorbereiten.

19

(145) Rafael hat das Hemd, das frisch gewaschen war, jemals lange getragen.

(146) Rafael hat kein Hemd, das frisch gewaschen war, jemals lange getragen.

(147) Rafael hat das Hemd, das frisch gewaschen war, schon lange getragen.

(148) Rafael hat kein Hemd, das frisch gewaschen war, schon lange getragen.

(149) Rafael hat das Hemd, das frisch gewaschen war, sehr lange getragen.

(150) Rafael hat kein Hemd, das frisch gewaschen war, sehr lange getragen.

(151) Rafael hat kein Hemd, das frisch gewaschen war, sehr lange besiegt.

(152) Rafael hat das Hemd, das frisch gewaschen war, sehr lange tragen.

20

(153) Amelie hat die Entscheidung, die gut bedacht war, jemals lange bereut.

(154) Amelie hat keine Entscheidung, die gut bedacht war, jemals lange bereut.

(155) Amelie hat die Entscheidung, die gut bedacht war, schon lange bereut.

(156) Amelie hat keine Entscheidung, die gut bedacht war, schon lange bereut.

(157) Amelie hat die Entscheidung, die gut bedacht war, sehr lange bereut.

(158) Amelie hat keine Entscheidung, die gut bedacht war, sehr lange bereut.

(159) Amelie hat keine Entscheidung, die gut bedacht war, sehr lange gewaschen.

(160) Amelie hat die Entscheidung, die gut bedacht war, sehr lange bereuen.

21

(161) Sebastian hat den Gegner, der im Ring trainierte, jemals oft besiegt.

(162) Sebastian hat keinen Gegner, der im Ring trainierte, jemals oft besiegt.

(163) Sebastian hat den Gegner, der im Ring trainierte, schon oft besiegt.

(164) Sebastian hat keinen Gegner, der im Ring trainierte, schon oft besiegt.

(165) Sebastian hat den Gegner, der im Ring trainierte, sehr oft besiegt.

(166) Sebastian hat keinen Gegner, der im Ring trainierte, sehr oft besiegt.

(167) Sebastian hat den Gegner, der im Ring trainierte, sehr oft geöffnet.

(168) Sebastian hat keinen Gegner, der im Ring trainierte, sehr oft besiegen.

22

(169) Denise hat das Lied, das ihrer Mutter gefiel, jemals oft gesungen.

(170) Denise hat kein Lied, das ihrer Mutter gefiel, jemals oft gesungen.

(171) Denise hat das Lied, das ihrer Mutter gefiel, schon oft gesungen.

(172) Denise hat kein Lied, das ihrer Mutter gefiel, schon oft gesungen.

(173) Denise hat das Lied, das ihrer Mutter gefiel, sehr oft gesungen.

(174) Denise hat kein Lied, das ihrer Mutter gefiel, sehr oft gesungen.

(175) Denise hat das Lied, das ihrer Mutter gefiel, sehr oft beobachtet.

(176) Denise hat kein Lied, das ihrer Mutter gefiel, sehr oft singen.

23

(177) Paul hat das Brot, das viel Weißmehl enthielt, jemals oft gekauft.

(178) Paul hat kein Brot, das viel Weißmehl enthielt, jemals oft gekauft.

(179) Paul hat das Brot, das viel Weißmehl enthielt, schon oft gekauft.

(180) Paul hat kein Brot, das viel Weißmehl enthielt, schon oft gekauft.

(181) Paul hat das Brot, das viel Weißmehl enthielt, sehr oft gekauft.

(182) Paul hat kein Brot, das viel Weißmehl enthielt, sehr oft gekauft.

(183) Paul hat das Brot, das viel Weißmehl enthielt, sehr oft geputzt.

(184) Paul hat kein Brot, das viel Weißmehl enthielt, sehr oft kaufen.

24

(185) Barbara hat das Spiel, das alle Kinder mochten, jemals lange gespielt.

(186) Barbara hat kein Spiel, das alle Kinder mochten, jemals lange gespielt.

(187) Barbara hat das Spiel, das alle Kinder mochten, schon lange gespielt.

(188) Barbara hat kein Spiel, das alle Kinder mochten, schon lange gespielt.

(189) Barbara hat das Spiel, das alle Kinder mochten, sehr lange gespielt.

(190) Barbara hat kein Spiel, das alle Kinder mochten, sehr lange gespielt.

(191) Barbara hat das Spiel, das alle Kinder mochten, sehr lange bewohnt.

(192) Barbara hat kein Spiel, das alle Kinder mochten, sehr lange spielen.

25

(193) Nick hat das Fahrrad, das kaputte Lichter hatte, jemals oft gefahren.

(194) Nick hat kein Fahrrad, das kaputte Lichter hatte, jemals oft gefahren.

(195) Nick hat das Fahrrad, das kaputte Lichter hatte, schon oft gefahren.

(196) Nick hat kein Fahrrad, das kaputte Lichter hatte, schon oft gefahren.

(197) Nick hat das Fahrrad, das kaputte Lichter hatte, sehr oft gefahren.

(198) Nick hat kein Fahrrad, das kaputte Lichter hatte, sehr oft gefahren.

(199) Nick hat das Fahrrad, das kaputte Lichter hatte, sehr oft unterrichtet.

(200) Nick hat kein Fahrrad, das kaputte Lichter hatte, sehr oft fahren.

26

(201) Christina hat das Geld, das ihr geschenkt wurde, jemals lange gespart.

(202) Christina hat kein Geld, das ihr geschenkt wurde, jemals lange gespart.

(203) Christina hat das Geld, das ihr geschenkt wurde, schon lange gespart.

(204) Christina hat kein Geld, das ihr geschenkt wurde, schon lange gespart.

(205) Christina hat das Geld, das ihr geschenkt wurde, sehr lange gespart.

(206) Christina hat kein Geld, das ihr geschenkt wurde, sehr lange gespart.

(207) Christina hat das Geld, das ihr geschenkt wurde, sehr lange bewohnt.

(208) Christina hat kein Geld, das ihr geschenkt wurde, sehr lange sparen.

27

(209) Markus hat das Haus, das kaputte Fenster hatte, jemals lange renoviert.

(210) Markus hat kein Haus, das kaputte Fenster hatte, jemals lange renoviert.

(211) Markus hat das Haus, das kaputte Fenster hatte, schon lange renoviert.

(212) Markus hat kein Haus, das kaputte Fenster hatte, schon lange renoviert.

(213) Markus hat das Haus, das kaputte Fenster hatte, sehr lange renoviert.

(214) Markus hat kein Haus, das kaputte Fenster hatte, sehr lange renoviert.

(215) Markus hat das Haus, das kaputte Fenster hatte, sehr lange getragen.

(216) Markus hat kein Haus, das kaputte Fenster hatte, sehr lange renovieren.

28

(217) Greta hat die Bluse, die aus Seide war, jemals oft gewaschen.

(218) Greta hat keine Bluse, die aus Seide war, jemals oft gewaschen.

(219) Greta hat die Bluse, die aus Seide war, schon oft gewaschen.

(220) Greta hat keine Bluse, die aus Seide war, schon oft gewaschen.

(221) Greta hat die Bluse, die aus Seide war, sehr oft gewaschen.

(222) Greta hat keine Bluse, die aus Seide war, sehr oft gewaschen.

(223) Greta hat die Bluse, die aus Seide war, sehr oft gebacken.

(224) Greta hat keine Bluse, die aus Seide war, sehr oft waschen.

29

(225) Fabian hat die Schokolade, die aus Deutschland stammte, jemals oft gegessen.

(226) Fabian hat keine Schokolade, die aus Deutschland stammte, jemals oft gegessen.

(227) Fabian hat die Schokolade, die aus Deutschland stammte, schon oft gegessen.

(228) Fabian hat keine Schokolade, die aus Deutschland stammte, schon oft gegessen.

(229) Fabian hat die Schokolade, die aus Deutschland stammte, sehr oft gegessen.

(230) Fabian hat keine Schokolade, die aus Deutschland stammte, sehr oft gegessen.

(231) Fabian hat die Schokolade, die aus Deutschland stammte, sehr oft belogen.

(232) Fabian hat keine Schokolade, die aus Deutschland stammte, sehr oft essen.

30

(233) Isabel hat das Auto, das viel Benzin verbrauchte, jemals oft gefahren.

(234) Isabel hat kein Auto, das viel Benzin verbrauchte, jemals oft gefahren.

(235) Isabel hat das Auto, das viel Benzin verbrauchte, schon oft gefahren.

(236) Isabel hat kein Auto, das viel Benzin verbrauchte, schon oft gefahren.

(237) Isabel hat das Auto, das viel Benzin verbrauchte, sehr oft gefahren.

(238) Isabel hat kein Auto, das viel Benzin verbrauchte, sehr oft gefahren.

(239) Isabel hat das Auto, das viel Benzin verbrauchte, sehr oft unterrichtet.

(240) Isabel hat kein Auto, das viel Benzin verbrauchte, sehr oft fahren.

31

(241) Helen hat den Vogel, der ein Nest baute, jemals lange beobachtet.

(242) Helen hat keinen Vogel, der ein Nest baute, jemals lange beobachtet.

(243) Helen hat den Vogel, der ein Nest baute, schon lange beobachtet.

(244) Helen hat keinen Vogel, der ein Nest baute, schon lange beobachtet.

(245) Helen hat den Vogel, der ein Nest baute, sehr lange beobachtet.

(246) Helen hat keinen Vogel, der ein Nest baute, sehr lange beobachtet.

(247) Helen hat den Vogel, der ein Nest baute, sehr lange getrunken.

(248) Helen hat keinen Vogel, der ein Nest baute, sehr lange beobachten.

32

(249) Bernd hat den Patienten, der häufig krank war, jemals lange untersucht.

(250) Bernd hat keinen Patienten, der häufig krank war, jemals lange untersucht.

(251) Bernd hat den Patienten, der häufig krank war, schon lange untersucht.

(252) Bernd hat keinen Patienten, der häufig krank war, schon lange untersucht.

(253) Bernd hat den Patienten, der häufig krank war, sehr lange untersucht.

(254) Bernd hat keinen Patienten, der häufig krank war, sehr lange untersucht.

(255) Bernd hat den Patienten, der häufig krank war, sehr lange ausgeliehen.

(256) Bernd hat keinen Patienten, der häufig krank war, sehr lange untersuchen.

33

(257) Eva hat die Tante, die in England lebte, jemals oft besucht.

(258) Eva hat keine Tante, die in England lebte, jemals oft besucht.

(259) Eva hat die Tante, die in England lebte, schon oft besucht.

(260) Eva hat keine Tante, die in England lebte, schon oft besucht.

(261) Eva hat die Tante, die in England lebte, sehr oft besucht.

(262) Eva hat keine Tante, die in England lebte, sehr oft besucht.

(263) Eva hat die Tante, die in England lebte, sehr oft gespielt.

(264) Eva hat keine Tante, die in England lebte, sehr oft besuchen.

34

(265) Anton hat den Schüler, der sich schlecht benahm, jemals lange unterrichtet.

(266) Anton hat keinen Schüler, der sich schlecht benahm, jemals lange unterrichtet.

(267) Anton hat den Schüler, der sich schlecht benahm, schon lange unterrichtet.

(268) Anton hat keinen Schüler, der sich schlecht benahm, schon lange unterrichtet.

(269) Anton hat den Schüler, der sich schlecht benahm, sehr lange unterrichtet.

(270) Anton hat keinen Schüler, der sich schlecht benahm, sehr lange unterrichtet.

(271) Anton hat den Schüler, der sich schlecht benahm, sehr lange aufgeräumt.

(272) Anton hat keinen Schüler, der sich schlecht benahm, sehr lange unterrichten.

35

(273) Paula hat den Saft, der viel Zucker enthielt, jemals oft getrunken.

(274) Paula hat keinen Saft, der viel Zucker enthielt, jemals oft getrunken.

(275) Paula hat den Saft, der viel Zucker enthielt, schon oft getrunken.

(276) Paula hat keinen Saft, der viel Zucker enthielt, schon oft getrunken.

(277) Paula hat den Saft, der viel Zucker enthielt, sehr oft getrunken.

(278) Paula hat keinen Saft, der viel Zucker enthielt, sehr oft getrunken.

(279) Paula hat den Saft, der viel Zucker enthielt, sehr oft geübt.

(280) Paula hat keinen Saft, der viel Zucker enthielt, sehr oft trinken.

36

(281) Walter hat das Auto, das schlechte Bremsen hatte, jemals lange gefahren.

(282) Walter hat kein Auto, das schlechte Bremsen hatte, jemals lange gefahren.

(283) Walter hat das Auto, das schlechte Bremsen hatte, schon lange gefahren.

(284) Walter hat kein Auto, das schlechte Bremsen hatte, schon lange gefahren.

(285) Walter hat das Auto, das schlechte Bremsen hatte, sehr lange gefahren.

(286) Walter hat kein Auto, das schlechte Bremsen hatte, sehr lange gefahren.

(287) Walter hat das Auto, das schlechte Bremsen hatte, sehr lange gespitzt.

(288) Walter hat kein Auto, das schlechte Bremsen hatte, sehr lange fahren.

37

(289) Viktoria hat den Freund, der weit entfernt lebte, jemals oft getroffen.

(290) Viktoria hat keinen Freund, der weit entfernt lebte, jemals oft getroffen.

(291) Viktoria hat den Freund, der weit entfernt lebte, schon oft getroffen.

(292) Viktoria hat keinen Freund, der weit entfernt lebte, schon oft getroffen.

(293) Viktoria hat den Freund, der weit entfernt lebte, sehr oft getroffen.

(294) Viktoria hat keinen Freund, der weit entfernt lebte, sehr oft getroffen.

(295) Viktoria hat den Freund, der weit entfernt lebte, sehr oft vorgetragen.

(296) Viktoria hat keinen Freund, der weit entfernt lebte, sehr oft treffen.

38

(297) Tanja hat die Tasche, die ihrer Schwester gehörte, jemals oft benutzt.

(298) Tanja hat keine Tasche, die ihrer Schwester gehörte, jemals oft benutzt.

(299) Tanja hat die Tasche, die ihrer Schwester gehörte, schon oft benutzt.

(300) Tanja hat keine Tasche, die ihrer Schwester gehörte, schon oft benutzt.

(301) Tanja hat die Tasche, die ihrer Schwester gehörte, sehr oft benutzt.

(302) Tanja hat keine Tasche, die ihrer Schwester gehörte, sehr oft benutzt.

(303) Tanja hat die Tasche, die ihrer Schwester gehörte, sehr oft unterstützt.

(304) Tanja hat keine Tasche, die ihrer Schwester gehörte, sehr oft benutzen.

39

(305) Florian hat die Hose, die große Löcher hatte, jemals lange getragen.

(306) Florian hat keine Hose, die große Löcher hatte, jemals lange getragen.

(307) Florian hat die Hose, die große Löcher hatte, schon lange getragen.

(308) Florian hat keine Hose, die große Löcher hatte, schon lange getragen.

(309) Florian hat die Hose, die große Löcher hatte, sehr lange getragen.

(310) Florian hat keine Hose, die große Löcher hatte, sehr lange getragen.

(311) Florian hat die Hose, die große Löcher hatte, sehr lange bereist.

(312) Florian hat keine Hose, die große Löcher hatte, sehr lange tragen.

40

(313) Holger hat den Supermarkt, der italienische Nudeln verkaufte, jemals oft besucht.

(314) Holger hat keinen Supermarkt, der italienische Nudeln verkaufte, jemals oft besucht.

(315) Holger hat den Supermarkt, der italienische Nudeln verkaufte, schon oft besucht.

(316) Holger hat keinen Supermarkt, der italienische Nudeln verkaufte, schon oft besucht.

(317) Holger hat den Supermarkt, der italienische Nudeln verkaufte, sehr oft besucht.

(318) Holger hat keinen Supermarkt, der italienische Nudeln verkaufte, sehr oft besucht.

(319) Holger hat den Supermarkt, der italienische Nudeln verkaufte, sehr oft gegrüßt.

(320) Holger hat keinen Supermarkt, der italienische Nudeln verkaufte, sehr oft besuchen.

41

(321) Johanna hat das Buch, das ein Liebesroman war, jemals lange gelesen.

(322) Johanna hat kein Buch, das ein Liebesroman war, jemals lange gelesen.

(323) Johanna hat das Buch, das ein Liebesroman war, schon lange gelesen.

(324) Johanna hat kein Buch, das ein Liebesroman war, schon lange gelesen.

(325) Johanna hat das Buch, das ein Liebesroman war, sehr lange gelesen.

(326) Johanna hat kein Buch, das ein Liebesroman war, sehr lange gelesen.

(327) Johanna hat kein Buch, das ein Liebesroman war, jemals lange gegessen.

(328) Johanna hat kein Buch, das ein Liebesroman war, jemals lange lesen.

42

(329) Volker hat das Haus, das hohe Nebenkosten erzeugte, jemals lange bewohnt.

(330) Volker hat kein Haus, das hohe Nebenkosten erzeugte, jemals lange bewohnt.

(331) Volker hat das Haus, das hohe Nebenkosten erzeugte, schon lange bewohnt.

(332) Volker hat kein Haus, das hohe Nebenkosten erzeugte, schon lange bewohnt.

(333) Volker hat das Haus, das hohe Nebenkosten erzeugte, sehr lange bewohnt.

(334) Volker hat kein Haus, das hohe Nebenkosten erzeugte, sehr lange bewohnt.

(335) Volker hat kein Haus, das hohe Nebenkosten erzeugte, jemals lange bedient.

(336) Volker hat kein Haus, das hohe Nebenkosten erzeugte, jemals lange bewohnen.

43

(337) Katharina hat den Kuchen, der viele Früchte enthielt, jemals oft gebacken.

(338) Katharina hat keinen Kuchen, der viele Früchte enthielt, jemals oft gebacken.

(339) Katharina hat den Kuchen, der viele Früchte enthielt, schon oft gebacken.

(340) Katharina hat keinen Kuchen, der viele Früchte enthielt, schon oft gebacken.

(341) Katharina hat den Kuchen, der viele Früchte enthielt, sehr oft gebacken.

(342) Katharina hat keinen Kuchen, der viele Früchte enthielt, sehr oft gebacken.

(343) Katharina hat keinen Kuchen, der viele Früchte enthielt, jemals oft gebügelt.

(344) Katharina hat keinen Kuchen, der viele Früchte enthielt, jemals oft backen.

44

(345) Robert hat das Restaurant, das bayerische Gerichte anbot, jemals oft besucht.

(346) Robert hat kein Restaurant, das bayerische Gerichte anbot, jemals oft besucht.

(347) Robert hat das Restaurant, das bayerische Gerichte anbot, schon oft besucht.

(348) Robert hat kein Restaurant, das bayerische Gerichte anbot, schon oft besucht.

(349) Robert hat das Restaurant, das bayerische Gerichte anbot, sehr oft besucht.

(350) Robert hat kein Restaurant, das bayerische Gerichte anbot, sehr oft besucht.

(351) Robert hat kein Restaurant, das bayerische Gerichte anbot, jemals oft gelutscht.

(352) Robert hat kein Restaurant, das bayerische Gerichte anbot, jemals oft besuchen.

45

(353) Rebekka hat den Rekord, der international bekannt war, jemals lange gehalten.

(354) Rebekka hat keinen Rekord, der international bekannt war, jemals lange gehalten.

(355) Rebekka hat den Rekord, der international bekannt war, schon lange gehalten.

(356) Rebekka hat keinen Rekord, der international bekannt war, schon lange gehalten.

(357) Rebekka hat den Rekord, der international bekannt war, sehr lange gehalten.

(358) Rebekka hat keinen Rekord, der international bekannt war, sehr lange gehalten.

(359) Rebekka hat keinen Rekord, der international bekannt war, jemals lange angebaut.

(360) Rebekka hat keinen Rekord, der international bekannt war, jemals lange halten.

46

(361) Nina hat den Käse, der sehr stark riecht, jemals oft gekauft.

(362) Nina hat keinen Käse, der sehr stark riecht, jemals oft gekauft.

(363) Nina hat den Käse, der sehr stark riecht, schon oft gekauft.

(364) Nina hat keinen Käse, der sehr stark riecht, schon oft gekauft.

(365) Nina hat den Käse, der sehr stark riecht, sehr oft gekauft.

(366) Nina hat keinen Käse, der sehr stark riecht, sehr oft gekauft.

(367) Nina hat keinen Käse, der sehr stark riecht, jemals oft angerufen.

(368) Nina hat keinen Käse, der sehr stark riecht, jemals oft kaufen.

47

(369) Otto hat die Textstelle, die er zitieren wollte, jemals lange gesucht.

(370) Otto hat keine Textstelle, die er zitieren wollte, jemals lange gesucht.

(371) Otto hat die Textstelle, die er zitieren wollte, schon lange gesucht.

(372) Otto hat keine Textstelle, die er zitieren wollte, schon lange gesucht.

(373) Otto hat die Textstelle, die er zitieren wollte, sehr lange gesucht.

(374) Otto hat keine Textstelle, die er zitieren wollte, sehr lange gesucht.

(375) Otto hat keine Textstelle, die er zitieren wollte, jemals lange betrieben.

(376) Otto hat keine Textstelle, die er zitieren wollte, jemals lange suchen.

48

(377) Nils hat das Mädchen, das blaue Augen hatte, jemals lange gemocht.

(378) Nils hat kein Mädchen, das blaue Augen hatte, jemals lange gemocht.

(379) Nils hat das Mädchen, das blaue Augen hatte, schon lange gemocht.

(380) Nils hat kein Mädchen, das blaue Augen hatte, schon lange gemocht.

(381) Nils hat das Mädchen, das blaue Augen hatte, sehr lange gemocht.

(382) Nils hat kein Mädchen, das blaue Augen hatte, sehr lange gemocht.

(383) Nils hat kein Mädchen, das blaue Augen hatte, jemals lange vermietet.

(384) Nils hat kein Mädchen, das blaue Augen hatte, jemals lange mögen.

49

(385) Stefanie hat das Buch, das politische Themen behandelte, jemals oft gelesen.

(386) Stefanie hat kein Buch, das politische Themen behandelte, jemals oft gelesen.

(387) Stefanie hat das Buch, das politische Themen behandelte, schon oft gelesen.

(388) Stefanie hat kein Buch, das politische Themen behandelte, schon oft gelesen.

(389) Stefanie hat das Buch, das politische Themen behandelte, sehr oft gelesen.

(390) Stefanie hat kein Buch, das politische Themen behandelte, sehr oft gelesen.

(391) Stefanie hat kein Buch, das politische Themen behandelte, jemals oft befragt.

(392) Stefanie hat kein Buch, das politische Themen behandelte, jemals oft lesen.

50

(393) Klara hat die Freundin, die ihr blind vertraute, jemals oft belogen.

(394) Klara hat keine Freundin, die ihr blind vertraute, jemals oft belogen.

(395) Klara hat die Freundin, die ihr blind vertraute, schon oft belogen.

(396) Klara hat keine Freundin, die ihr blind vertraute, schon oft belogen.

(397) Klara hat die Freundin, die ihr blind vertraute, sehr oft belogen.

(398) Klara hat keine Freundin, die ihr blind vertraute, sehr oft belogen.

(399) Klara hat keine Freundin, die ihr blind vertraute, jemals oft repariert.

(400) Klara hat keine Freundin, die ihr blind vertraute, jemals oft belügen.

51

(401) Daniel hat den Bus, der zur Schule fuhr, jemals oft verpasst.

(402) Daniel hat keinen Bus, der zur Schule fuhr, jemals oft verpasst.

(403) Daniel hat den Bus, der zur Schule fuhr, schon oft verpasst.

(404) Daniel hat keinen Bus, der zur Schule fuhr, schon oft verpasst.

(405) Daniel hat den Bus, der zur Schule fuhr, sehr oft verpasst.

(406) Daniel hat keinen Bus, der zur Schule fuhr, sehr oft verpasst.

(407) Daniel hat keinen Bus, der zur Schule fuhr, jemals oft gegossen.

(408) Daniel hat keinen Bus, der zur Schule fuhr, jemals oft verpassen.

52

(409) Werner hat den Vortrag, der kritische Themen behandelte, jemals oft gehalten.

(410) Werner hat keinen Vortrag, der kritische Themen behandelte, jemals oft gehalten.

(411) Werner hat den Vortrag, der kritische Themen behandelte, schon oft gehalten.

(412) Werner hat keinen Vortrag, der kritische Themen behandelte, schon oft gehalten.

(413) Werner hat den Vortrag, der kritische Themen behandelte, sehr oft gehalten.

(414) Werner hat keinen Vortrag, der kritische Themen behandelte, sehr oft gehalten.

(415) Werner hat keinen Vortrag, der kritische Themen behandelte, jemals oft gereinigt.

(416) Werner hat keinen Vortrag, der kritische Themen behandelte, jemals oft halten.

53

(417) Simone hat das Museum, das moderne Kunst ausstellte, jemals oft besucht.

(418) Simone hat kein Museum, das moderne Kunst ausstellte, jemals oft besucht.

(419) Simone hat das Museum, das moderne Kunst ausstellte, schon oft besucht.

(420) Simone hat kein Museum, das moderne Kunst ausstellte, schon oft besucht.

(421) Simone hat das Museum, das moderne Kunst ausstellte, sehr oft besucht.

(422) Simone hat kein Museum, das moderne Kunst ausstellte, sehr oft besucht.

(423) Simone hat kein Museum, das moderne Kunst ausstellte, jemals oft erzählt.

(424) Simone hat kein Museum, das moderne Kunst ausstellte, jemals oft besuchen.

54

(425) Torsten hat das Fahrrad, das seinem Freund gehörte, jemals oft ausgeliehen.

(426) Torsten hat kein Fahrrad, das seinem Freund gehörte, jemals oft ausgeliehen.

(427) Torsten hat das Fahrrad, das seinem Freund gehörte, schon oft ausgeliehen.

(428) Torsten hat kein Fahrrad, das seinem Freund gehörte, schon oft ausgeliehen.

(429) Torsten hat das Fahrrad, das seinem Freund gehörte, sehr oft ausgeliehen.

(430) Torsten hat kein Fahrrad, das seinem Freund gehörte, sehr oft ausgeliehen.

(431) Torsten hat kein Fahrrad, das seinem Freund gehörte, jemals oft gespült.

(432) Torsten hat kein Fahrrad, das seinem Freund gehörte, jemals oft ausleihen.

55

(433) Vera hat den Künstler, den sie persönlich kannte, jemals lange unterstützt.

(434) Vera hat keinen Künstler, den sie persönlich kannte, jemals lange unterstützt.

(435) Vera hat den Künstler, den sie persönlich kannte, schon lange unterstützt.

(436) Vera hat keinen Künstler, den sie persönlich kannte, schon lange unterstützt.

(437) Vera hat den Künstler, den sie persönlich kannte, sehr lange unterstützt.

(438) Vera hat keinen Künstler, den sie persönlich kannte, sehr lange unterstützt.

(439) Vera hat keinen Künstler, den sie persönlich kannte, jemals lange zubereitet.

(440) Vera hat keinen Künstler, den sie persönlich kannte, jemals lange unterstützen.

56

(441) Philipp hat das Hotel, das fünf Sterne hatte, jemals lange geführt.

(442) Philipp hat kein Hotel, das fünf Sterne hatte, jemals lange geführt.

(443) Philipp hat das Hotel, das fünf Sterne hatte, schon lange geführt.

(444) Philipp hat kein Hotel, das fünf Sterne hatte, schon lange geführt.

(445) Philipp hat das Hotel, das fünf Sterne hatte, sehr lange geführt.

(446) Philipp hat kein Hotel, das fünf Sterne hatte, sehr lange geführt.

(447) Philipp hat kein Hotel, das fünf Sterne hatte, jemals lange gestreichelt.

(448) Philipp hat kein Hotel, das fünf Sterne hatte, jemals lange führen.

57

(449) Tamara hat den Film, der viele Preise bekam, jemals oft gesehen.

(450) Tamara hat keinen Film, der viele Preise bekam, jemals oft gesehen.

(451) Tamara hat den Film, der viele Preise bekam, schon oft gesehen.

(452) Tamara hat keinen Film, der viele Preise bekam, schon oft gesehen.

(453) Tamara hat den Film, der viele Preise bekam, sehr oft gesehen.

(454) Tamara hat keinen Film, der viele Preise bekam, sehr oft gesehen.

(455) Tamara hat keinen Film, der viele Preise bekam, jemals oft gelüftet.

(456) Tamara hat keinen Film, der viele Preise bekam, jemals oft sehen.

58

(457) Moritz hat die Tablette, die gegen Kopfschmerzen wirkte, jemals oft genommen.

(458) Moritz hat keine Tablette, die gegen Kopfschmerzen wirkte, jemals oft genommen.

(459) Moritz hat die Tablette, die gegen Kopfschmerzen wirkte, schon oft genommen.

(460) Moritz hat keine Tablette, die gegen Kopfschmerzen wirkte, schon oft genommen.

(461) Moritz hat die Tablette, die gegen Kopfschmerzen wirkte, sehr oft genommen.

(462) Moritz hat keine Tablette, die gegen Kopfschmerzen wirkte, sehr oft genommen.

(463) Moritz hat keine Tablette, die gegen Kopfschmerzen wirkte, jemals oft erlernt.

(464) Moritz hat keine Tablette, die gegen Kopfschmerzen wirkte, jemals oft nehmen.

59

(465) Jens hat den Senioren, der viele Geschichten erzählte, jemals lange gepflegt.

(466) Jens hat keinen Senioren, der viele Geschichten erzählte, jemals lange gepflegt.

(467) Jens hat den Senioren, der viele Geschichten erzählte, schon lange gepflegt.

(468) Jens hat keinen Senioren, der viele Geschichten erzählte, schon lange gepflegt.

(469) Jens hat den Senioren, der viele Geschichten erzählte, sehr lange gepflegt.

(470) Jens hat keinen Senioren, der viele Geschichten erzählte, sehr lange gepflegt.

(471) Jens hat keinen Senioren, der viele Geschichten erzählte, jemals lange bestellt.

(472) Jens hat keinen Senioren, der viele Geschichten erzählte, jemals lange pflegen.

60

(473) Melissa hat das Kleid, das sie geschenkt bekam, jemals oft getragen.

(474) Melissa hat kein Kleid, das sie geschenkt bekam, jemals oft getragen.

(475) Melissa hat das Kleid, das sie geschenkt bekam, schon oft getragen.

(476) Melissa hat kein Kleid, das sie geschenkt bekam, schon oft getragen.

(477) Melissa hat das Kleid, das sie geschenkt bekam, sehr oft getragen.

(478) Melissa hat kein Kleid, das sie geschenkt bekam, sehr oft getragen.

(479) Melissa hat kein Kleid, das sie geschenkt bekam, jemals oft abgesagt.

(480) Melissa hat kein Kleid, das sie geschenkt bekam, jemals oft tragen.

61

(481) Gustav hat das Geheimnis, das ihm erzählt wurde, jemals lange bewahrt.

(482) Gustav hat kein Geheimnis, das ihm erzählt wurde, jemals lange bewahrt.

(483) Gustav hat das Geheimnis, das ihm erzählt wurde, schon lange bewahrt.

(484) Gustav hat kein Geheimnis, das ihm erzählt wurde, schon lange bewahrt.

(485) Gustav hat das Geheimnis, das ihm erzählt wurde, sehr lange bewahrt.

(486) Gustav hat kein Geheimnis, das ihm erzählt wurde, sehr lange bewahrt.

(487) Gustav hat kein Geheimnis, das ihm erzählt wurde, jemals lange abgesagt.

(488) Gustav hat kein Geheimnis, das ihm erzählt wurde, jemals lange bewahren.

62

(489) Konstanze hat die Nachbarin, die morgens unfreundlich war, jemals oft gegrüßt.

(490) Konstanze hat keine Nachbarin, die morgens unfreundlich war, jemals oft gegrüßt.

(491) Konstanze hat die Nachbarin, die morgens unfreundlich war, schon oft gegrüßt.

(492) Konstanze hat keine Nachbarin, die morgens unfreundlich war, schon oft gegrüßt.

(493) Konstanze hat die Nachbarin, die morgens unfreundlich war, sehr oft gegrüßt.

(494) Konstanze hat keine Nachbarin, die morgens unfreundlich war, sehr oft gegrüßt.

(495) Konstanze hat keine Nachbarin, die morgens unfreundlich war, jemals oft geplant.

(496) Konstanze hat keine Nachbarin, die morgens unfreundlich war, jemals oft grüßen.

63

(497) Dennis hat das Zimmer, das seinen Kindern gehörte, jemals oft aufgeräumt.

(498) Dennis hat kein Zimmer, das seinen Kindern gehörte, jemals oft aufgeräumt.

(499) Dennis hat das Zimmer, das seinen Kindern gehörte, schon oft aufgeräumt.

(500) Dennis hat kein Zimmer, das seinen Kindern gehörte, schon oft aufgeräumt.

(501) Dennis hat das Zimmer, das seinen Kindern gehörte, sehr oft aufgeräumt.

(502) Dennis hat kein Zimmer, das seinen Kindern gehörte, sehr oft aufgeräumt.

(503) Dennis hat kein Zimmer, das seinen Kindern gehörte, jemals oft gefahren.

(504) Dennis hat kein Zimmer, das seinen Kindern gehörte, jemals oft aufräumen.

64

(505) Andre hat den Bleistift, der besonders schön war, jemals oft gespitzt.

(506) Andre hat keinen Bleistift, der besonders schön war, jemals oft gespitzt.

(507) Andre hat den Bleistift, der besonders schön war, schon oft gespitzt.

(508) Andre hat keinen Bleistift, der besonders schön war, schon oft gespitzt.

(509) Andre hat den Bleistift, der besonders schön war, sehr oft gespitzt.

(510) Andre hat keinen Bleistift, der besonders schön war, sehr oft gespitzt.

(511) Andre hat keinen Bleistift, der besonders schön war, jemals oft überquert.

(512) Andre hat keinen Bleistift, der besonders schön war, jemals oft spitzen.

65

(513) Iris hat den Wein, der besonders teuer war, jemals oft getrunken.

(514) Iris hat keinen Wein, der besonders teuer war, jemals oft getrunken.

(515) Iris hat den Wein, der besonders teuer war, schon oft getrunken.

(516) Iris hat keinen Wein, der besonders teuer war, schon oft getrunken.

(517) Iris hat den Wein, der besonders teuer war, sehr oft getrunken.

(518) Iris hat keinen Wein, der besonders teuer war, sehr oft getrunken.

(519) Iris hat keinen Wein, der besonders teuer war, jemals oft geärgert.

(520) Iris hat keinen Wein, der besonders teuer war, jemals oft trinken.

66

(521) Tim hat das Instrument, das viele Tasten hatte, jemals oft geübt.

(522) Tim hat kein Instrument, das viele Tasten hatte, jemals oft geübt.

(523) Tim hat das Instrument, das viele Tasten hatte, schon oft geübt.

(524) Tim hat kein Instrument, das viele Tasten hatte, schon oft geübt.

(525) Tim hat das Instrument, das viele Tasten hatte, sehr oft geübt.

(526) Tim hat kein Instrument, das viele Tasten hatte, sehr oft geübt.

(527) Tim hat kein Instrument, das viele Tasten hatte, jemals oft betreut.

(528) Tim hat kein Instrument, das viele Tasten hatte, jemals oft üben.

67

(529) Corinna hat den Beruf, der ihrer Gesundheit schadete, jemals lange gemocht.

(530) Corinna hat keinen Beruf, der ihrer Gesundheit schadete, jemals lange gemocht.

(531) Corinna hat den Beruf, der ihrer Gesundheit schadete, schon lange gemocht.

(532) Corinna hat keinen Beruf, der ihrer Gesundheit schadete, schon lange gemocht.

(533) Corinna hat den Beruf, der ihrer Gesundheit schadete, sehr lange gemocht.

(534) Corinna hat keinen Beruf, der ihrer Gesundheit schadete, sehr lange gemocht.

(535) Corinna hat keinen Beruf, der ihrer Gesundheit schadete, jemals lange geritten.

(536) Corinna hat keinen Beruf, der ihrer Gesundheit schadete, jemals lange mögen.

68

(537) Simon hat das Gedicht, das Goethe geschrieben hatte, jemals oft vorgetragen.

(538) Simon hat kein Gedicht, das Goethe geschrieben hatte, jemals oft vorgetragen.

(539) Simon hat das Gedicht, das Goethe geschrieben hatte, schon oft vorgetragen.

(540) Simon hat kein Gedicht, das Goethe geschrieben hatte, schon oft vorgetragen.

(541) Simon hat das Gedicht, das Goethe geschrieben hatte, sehr oft vorgetragen.

(542) Simon hat kein Gedicht, das Goethe geschrieben hatte, sehr oft vorgetragen.

(543) Simon hat kein Gedicht, das Goethe geschrieben hatte, jemals oft gegessen.

(544) Simon hat kein Gedicht, das Goethe geschrieben hatte, jemals oft vortragen.

69

(545) Britta hat das Land, das hohe Berge hatte, jemals oft bereist.

(546) Britta hat kein Land, das hohe Berge hatte, jemals oft bereist.

(547) Britta hat das Land, das hohe Berge hatte, schon oft bereist.

(548) Britta hat kein Land, das hohe Berge hatte, schon oft bereist.

(549) Britta hat das Land, das hohe Berge hatte, sehr oft bereist.

(550) Britta hat kein Land, das hohe Berge hatte, sehr oft bereist.

(551) Britta hat kein Land, das hohe Berge hatte, jemals oft gekocht.

(552) Britta hat kein Land, das hohe Berge hatte, jemals oft bereisen.

70

(553) Angela hat das Ergebnis, das eine Überraschung war, jemals lange bezweifelt.

(554) Angela hat kein Ergebnis, das eine Überraschung war, jemals lange bezweifelt.

(555) Angela hat das Ergebnis, das eine Überraschung war, schon lange bezweifelt.

(556) Angela hat kein Ergebnis, das eine Überraschung war, schon lange bezweifelt.

(557) Angela hat das Ergebnis, das eine Überraschung war, sehr lange bezweifelt.

(558) Angela hat kein Ergebnis, das eine Überraschung war, sehr lange bezweifelt.

(559) Angela hat kein Ergebnis, das eine Überraschung war, jemals lange gesungen.

(560) Angela hat kein Ergebnis, das eine Überraschung war, jemals lange bezweifeln.

71

(561) Dominik hat den Mitarbeiter, der gute Arbeit leistete, jemals oft gelobt.

(562) Dominik hat keinen Mitarbeiter, der gute Arbeit leistete, jemals oft gelobt.

(563) Dominik hat den Mitarbeiter, der gute Arbeit leistete, schon oft gelobt.

(564) Dominik hat keinen Mitarbeiter, der gute Arbeit leistete, schon oft gelobt.

(565) Dominik hat den Mitarbeiter, der gute Arbeit leistete, sehr oft gelobt.

(566) Dominik hat keinen Mitarbeiter, der gute Arbeit leistete, sehr oft gelobt.

(567) Dominik hat keinen Mitarbeiter, der gute Arbeit leistete, jemals oft gekauft.

(568) Dominik hat keinen Mitarbeiter, der gute Arbeit leistete, jemals oft loben.

72

(569) Emily hat den Mann, der im Gefängnis war, jemals lange gehasst.

(570) Emily hat keinen Mann, der im Gefängnis war, jemals lange gehasst.

(571) Emily hat den Mann, der im Gefängnis war, schon lange gehasst.

(572) Emily hat keinen Mann, der im Gefängnis war, schon lange gehasst.

(573) Emily hat den Mann, der im Gefängnis war, sehr lange gehasst.

(574) Emily hat keinen Mann, der im Gefängnis war, sehr lange gehasst.

(575) Emily hat keinen Mann, der im Gefängnis war, jemals lange renoviert.

(576) Emily hat keinen Mann, der im Gefängnis war, jemals lange hassen.

73

(577) Helga hat die Suppe, die viel Gemüse enthielt, jemals oft gegessen.

(578) Helga hat keine Suppe, die viel Gemüse enthielt, jemals oft gegessen.

(579) Helga hat die Suppe, die viel Gemüse enthielt, schon oft gegessen.

(580) Helga hat keine Suppe, die viel Gemüse enthielt, schon oft gegessen.

(581) Helga hat die Suppe, die viel Gemüse enthielt, sehr oft gegessen.

(582) Helga hat keine Suppe, die viel Gemüse enthielt, sehr oft gegessen.

(583) Helga hat keine Suppe, die viel Gemüse enthielt, jemals oft besiegt.

(584) Helga hat keine Suppe, die viel Gemüse enthielt, jemals oft essen.

74

(585) Imke hat die Geschichte, die ihr peinlich war, jemals oft erzählt.

(586) Imke hat keine Geschichte, die ihr peinlich war, jemals oft erzählt.

(587) Imke hat die Geschichte, die ihr peinlich war, schon oft erzählt.

(588) Imke hat keine Geschichte, die ihr peinlich war, schon oft erzählt.

(589) Imke hat die Geschichte, die ihr peinlich war, sehr oft erzählt.

(590) Imke hat keine Geschichte, die ihr peinlich war, sehr oft erzählt.

(591) Imke hat keine Geschichte, die ihr peinlich war, jemals oft getragen.

(592) Imke hat keine Geschichte, die ihr peinlich war, jemals oft erzählen.

75

(593) Matthias hat den Kunden, der viel Trinkgeld gab, jemals oft bedient.

(594) Matthias hat keinen Kunden, der viel Trinkgeld gab, jemals oft bedient.

(595) Matthias hat den Kunden, der viel Trinkgeld gab, schon oft bedient.

(596) Matthias hat keinen Kunden, der viel Trinkgeld gab, schon oft bedient.

(597) Matthias hat den Kunden, der viel Trinkgeld gab, sehr oft bedient.

(598) Matthias hat keinen Kunden, der viel Trinkgeld gab, sehr oft bedient.

(599) Matthias hat keinen Kunden, der viel Trinkgeld gab, jemals oft gesungen.

(600) Matthias hat keinen Kunden, der viel Trinkgeld gab, jemals oft bedienen.

76

(601) Karla hat das Hemd, das ihrem Mann gehörte, jemals oft gebügelt.

(602) Karla hat kein Hemd, das ihrem Mann gehörte, jemals oft gebügelt.

(603) Karla hat das Hemd, das ihrem Mann gehörte, schon oft gebügelt.

(604) Karla hat kein Hemd, das ihrem Mann gehörte, schon oft gebügelt.

(605) Karla hat das Hemd, das ihrem Mann gehörte, sehr oft gebügelt.

(606) Karla hat kein Hemd, das ihrem Mann gehörte, sehr oft gebügelt.

(607) Karla hat kein Hemd, das ihrem Mann gehörte, jemals oft getroffen.

(608) Karla hat kein Hemd, das ihrem Mann gehörte, jemals oft bügeln.

77

(609) Steffen hat den Autor, der spannende Bücher schrieb, jemals oft kritisiert.

(610) Steffen hat keinen Autor, der spannende Bücher schrieb, jemals oft kritisiert.

(611) Steffen hat den Autor, der spannende Bücher schrieb, schon oft kritisiert.

(612) Steffen hat keinen Autor, der spannende Bücher schrieb, schon oft kritisiert.

(613) Steffen hat den Autor, der spannende Bücher schrieb, sehr oft kritisiert.

(614) Steffen hat keinen Autor, der spannende Bücher schrieb, sehr oft kritisiert.

(615) Steffen hat keinen Autor, der spannende Bücher schrieb, jemals oft bewohnt.

(616) Steffen hat keinen Autor, der spannende Bücher schrieb, jemals oft kritisieren.

78

(617) Luise hat das Restaurant, das teure Speisen servierte, jemals lange betrieben.

(618) Luise hat kein Restaurant, das teure Speisen servierte, jemals lange betrieben.

(619) Luise hat das Restaurant, das teure Speisen servierte, schon lange betrieben.

(620) Luise hat kein Restaurant, das teure Speisen servierte, schon lange betrieben.

(621) Luise hat das Restaurant, das teure Speisen servierte, sehr lange betrieben.

(622) Luise hat kein Restaurant, das teure Speisen servierte, sehr lange betrieben.

(623) Luise hat kein Restaurant, das teure Speisen servierte, jemals lange gespielt.

(624) Luise hat kein Restaurant, das teure Speisen servierte, jemals lange betreiben.

79

(625) Maria hat das Bild, das nackte Menschen zeigte, jemals lange ausgestellt.

(626) Maria hat kein Bild, das nackte Menschen zeigte, jemals lange ausgestellt.

(627) Maria hat das Bild, das nackte Menschen zeigte, schon lange ausgestellt.

(628) Maria hat kein Bild, das nackte Menschen zeigte, schon lange ausgestellt.

(629) Maria hat das Bild, das nackte Menschen zeigte, sehr lange ausgestellt.

(630) Maria hat kein Bild, das nackte Menschen zeigte, sehr lange ausgestellt.

(631) Maria hat kein Bild, das nackte Menschen zeigte, jemals lange verpasst.

(632) Maria hat kein Bild, das nackte Menschen zeigte, jemals lange ausstellen.

80

(633) Sascha hat den Freund, der viel Aufmerksamkeit brauchte, jemals oft angerufen.

(634) Sascha hat keinen Freund, der viel Aufmerksamkeit brauchte, jemals oft angerufen.

(635) Sascha hat den Freund, der viel Aufmerksamkeit brauchte, schon oft angerufen.

(636) Sascha hat keinen Freund, der viel Aufmerksamkeit brauchte, schon oft angerufen.

(637) Sascha hat den Freund, der viel Aufmerksamkeit brauchte, sehr oft angerufen.

(638) Sascha hat keinen Freund, der viel Aufmerksamkeit brauchte, sehr oft angerufen.

(639) Sascha hat keinen Freund, der viel Aufmerksamkeit brauchte, jemals oft geübt.

(640) Sascha hat keinen Freund, der viel Aufmerksamkeit brauchte, jemals oft anrufen.

81

(641) Ramona hat die Apfelsorte, die viel Sonne benötigte, jemals lange angebaut.

(642) Ramona hat keine Apfelsorte, die viel Sonne benötigte, jemals lange angebaut.

(643) Ramona hat die Apfelsorte, die viel Sonne benötigte, schon lange angebaut.

(644) Ramona hat keine Apfelsorte, die viel Sonne benötigte, schon lange angebaut.

(645) Ramona hat die Apfelsorte, die viel Sonne benötigte, sehr lange angebaut.

(646) Ramona hat keine Apfelsorte, die viel Sonne benötigte, sehr lange angebaut.

(647) Ramona hat die Apfelsorte, die viel Sonne benötigte, schon lange betrieben.

(648) Ramona hat die Apfelsorte, die viel Sonne benötigte, schon lange anbauen.

82

(649) Nico hat die Wohnung, die kleine Zimmer hatte, jemals lange vermietet.

(650) Nico hat keine Wohnung, die kleine Zimmer hatte, jemals lange vermietet.

(651) Nico hat die Wohnung, die kleine Zimmer hatte, schon lange vermietet.

(652) Nico hat keine Wohnung, die kleine Zimmer hatte, schon lange vermietet.

(653) Nico hat die Wohnung, die kleine Zimmer hatte, sehr lange vermietet.

(654) Nico hat keine Wohnung, die kleine Zimmer hatte, sehr lange vermietet.

(655) Nico hat die Wohnung, die kleine Zimmer hatte, schon lange gelesen.

(656) Nico hat die Wohnung, die kleine Zimmer hatte, schon lange vermieten.

83

(657) Susanne hat das Messer, das rostige Stellen hatte, jemals lange benutzt.

(658) Susanne hat kein Messer, das rostige Stellen hatte, jemals lange benutzt.

(659) Susanne hat das Messer, das rostige Stellen hatte, schon lange benutzt.

(660) Susanne hat kein Messer, das rostige Stellen hatte, schon lange benutzt.

(661) Susanne hat das Messer, das rostige Stellen hatte, sehr lange benutzt.

(662) Susanne hat kein Messer, das rostige Stellen hatte, sehr lange benutzt.

(663) Susanne hat das Messer, das rostige Stellen hatte, schon lange gestreichelt.

(664) Susanne hat das Messer, das rostige Stellen hatte, schon lange benutzen.

84

(665) Tina hat das Bonbon, das extrem süß war, jemals lange gelutscht.

(666) Tina hat kein Bonbon, das extrem süß war, jemals lange gelutscht.

(667) Tina hat das Bonbon, das extrem süß war, schon lange gelutscht.

(668) Tina hat kein Bonbon, das extrem süß war, schon lange gelutscht.

(669) Tina hat das Bonbon, das extrem süß war, sehr lange gelutscht.

(670) Tina hat kein Bonbon, das extrem süß war, sehr lange gelutscht.

(671) Tina hat das Bonbon, das extrem süß war, schon lange gehört.

(672) Tina hat das Bonbon, das extrem süß war, schon lange lutschen.

85

(673) Lars hat den Arbeitgeber, der sich unfreundlich verhielt, jemals lange respektiert.

(674) Lars hat keinen Arbeitgeber, der sich unfreundlich verhielt, jemals lange respektiert.

(675) Lars hat den Arbeitgeber, der sich unfreundlich verhielt, schon lange respektiert.

(676) Lars hat keinen Arbeitgeber, der sich unfreundlich verhielt, schon lange respektiert.

(677) Lars hat den Arbeitgeber, der sich unfreundlich verhielt, sehr lange respektiert.

(678) Lars hat keinen Arbeitgeber, der sich unfreundlich verhielt, sehr lange respektiert.

(679) Lars hat den Arbeitgeber, der sich unfreundlich verhielt, schon lange repariert.

(680) Lars hat den Arbeitgeber, der sich unfreundlich verhielt, schon lange respektieren.

86

(681) Viola hat den Hund, der schmutziges Fell hatte, jemals oft gestreichelt.

(682) Viola hat keinen Hund, der schmutziges Fell hatte, jemals oft gestreichelt.

(683) Viola hat den Hund, der schmutziges Fell hatte, schon oft gestreichelt.

(684) Viola hat keinen Hund, der schmutziges Fell hatte, schon oft gestreichelt.

(685) Viola hat den Hund, der schmutziges Fell hatte, sehr oft gestreichelt.

(686) Viola hat keinen Hund, der schmutziges Fell hatte, sehr oft gestreichelt.

(687) Viola hat den Hund, der schmutziges Fell hatte, schon oft gefeiert.

(688) Viola hat den Hund, der schmutziges Fell hatte, schon oft streicheln.

87

(689) Kai hat das Land, das weit entfernt war, jemals oft bereist.

(690) Kai hat kein Land, das weit entfernt war, jemals oft bereist.

(691) Kai hat das Land, das weit entfernt war, schon oft bereist.

(692) Kai hat kein Land, das weit entfernt war, schon oft bereist.

(693) Kai hat das Land, das weit entfernt war, sehr oft bereist.

(694) Kai hat kein Land, das weit entfernt war, sehr oft bereist.

(695) Kai hat das Land, das weit entfernt war, schon oft geweckt.

(696) Kai hat das Land, das weit entfernt war, schon oft bereisen.

88

(697) Alina hat das Lied, das traurige Erinnerungen weckte, jemals oft gehört.

(698) Alina hat kein Lied, das traurige Erinnerungen weckte, jemals oft gehört.

(699) Alina hat das Lied, das traurige Erinnerungen weckte, schon oft gehört.

(700) Alina hat kein Lied, das traurige Erinnerungen weckte, schon oft gehört.

(701) Alina hat das Lied, das traurige Erinnerungen weckte, sehr oft gehört.

(702) Alina hat kein Lied, das traurige Erinnerungen weckte, sehr oft gehört.

(703) Alina hat das Lied, das traurige Erinnerungen weckte, schon oft befragt.

(704) Alina hat das Lied, das traurige Erinnerungen weckte, schon oft hören.

89

(705) Beate hat den Schauspieler, der nur Nebenrollen spielte, jemals lange bewundert.

(706) Beate hat keinen Schauspieler, der nur Nebenrollen spielte, jemals lange bewundert.

(707) Beate hat den Schauspieler, der nur Nebenrollen spielte, schon lange bewundert.

(708) Beate hat keinen Schauspieler, der nur Nebenrollen spielte, schon lange bewundert.

(709) Beate hat den Schauspieler, der nur Nebenrollen spielte, sehr lange bewundert.

(710) Beate hat keinen Schauspieler, der nur Nebenrollen spielte, sehr lange bewundert.

(711) Beate hat den Schauspieler, der nur Nebenrollen spielte, schon lange besessen.

(712) Beate hat den Schauspieler, der nur Nebenrollen spielte, schon lange bewundern.

90

(713) Finn hat den Computer, der einen Virus hatte, jemals lange repariert.

(714) Finn hat keinen Computer, der einen Virus hatte, jemals lange repariert.

(715) Finn hat den Computer, der einen Virus hatte, schon lange repariert.

(716) Finn hat keinen Computer, der einen Virus hatte, schon lange repariert.

(717) Finn hat den Computer, der einen Virus hatte, sehr lange repariert.

(718) Finn hat keinen Computer, der einen Virus hatte, sehr lange repariert.

(719) Finn hat den Computer, der einen Virus hatte, schon lange betrogen.

(720) Finn hat den Computer, der einen Virus hatte, schon lange reparieren.

91

(721) Doris hat das Buch, das viele Seiten hatte, jemals oft gelesen.

(722) Doris hat kein Buch, das viele Seiten hatte, jemals oft gelesen.

(723) Doris hat das Buch, das viele Seiten hatte, schon oft gelesen.

(724) Doris hat kein Buch, das viele Seiten hatte, schon oft gelesen.

(725) Doris hat das Buch, das viele Seiten hatte, sehr oft gelesen.

(726) Doris hat kein Buch, das viele Seiten hatte, sehr oft gelesen.

(727) Doris hat das Buch, das viele Seiten hatte, schon oft abgesagt.

(728) Doris hat das Buch, das viele Seiten hatte, schon oft lesen.

92

(729) Arthur hat den Zeugen, der wichtige Informationen hatte, jemals lange befragt.

(730) Arthur hat keinen Zeugen, der wichtige Informationen hatte, jemals lange befragt.

(731) Arthur hat den Zeugen, der wichtige Informationen hatte, schon lange befragt.

(732) Arthur hat keinen Zeugen, der wichtige Informationen hatte, schon lange befragt.

(733) Arthur hat den Zeugen, der wichtige Informationen hatte, sehr lange befragt.

(734) Arthur hat keinen Zeugen, der wichtige Informationen hatte, sehr lange befragt.

(735) Arthur hat den Zeugen, der wichtige Informationen hatte, schon lange gespült.

(736) Arthur hat den Zeugen, der wichtige Informationen hatte, schon lange befragen.

93

(737) Elena hat den Nachtisch, der viele Kalorien hatte, jemals oft gegessen.

(738) Elena hat keinen Nachtisch, der viele Kalorien hatte, jemals oft gegessen.

(739) Elena hat den Nachtisch, der viele Kalorien hatte, schon oft gegessen.

(740) Elena hat keinen Nachtisch, der viele Kalorien hatte, schon oft gegessen.

(741) Elena hat den Nachtisch, der viele Kalorien hatte, sehr oft gegessen.

(742) Elena hat keinen Nachtisch, der viele Kalorien hatte, sehr oft gegessen.

(743) Elena hat den Nachtisch, der viele Kalorien hatte, schon oft gelüftet.

(744) Elena hat den Nachtisch, der viele Kalorien hatte, schon oft essen.

94

(745) David hat die Versicherung, die hohe Preise forderte, jemals oft betrogen.

(746) David hat keine Versicherung, die hohe Preise forderte, jemals oft betrogen.

(747) David hat die Versicherung, die hohe Preise forderte, schon oft betrogen.

(748) David hat keine Versicherung, die hohe Preise forderte, schon oft betrogen.

(749) David hat die Versicherung, die hohe Preise forderte, sehr oft betrogen.

(750) David hat keine Versicherung, die hohe Preise forderte, sehr oft betrogen.

(751) David hat die Versicherung, die hohe Preise forderte, schon oft gereinigt.

(752) David hat die Versicherung, die hohe Preise forderte, schon oft betrügen.

95

(753) Elisabeth hat das Medikament, das ihr Arzt empfahl, jemals lange genommen.

(754) Elisabeth hat kein Medikament, das ihr Arzt empfahl, jemals lange genommen.

(755) Elisabeth hat das Medikament, das ihr Arzt empfahl, schon lange genommen.

(756) Elisabeth hat kein Medikament, das ihr Arzt empfahl, schon lange genommen.

(757) Elisabeth hat das Medikament, das ihr Arzt empfahl, sehr lange genommen.

(758) Elisabeth hat kein Medikament, das ihr Arzt empfahl, sehr lange genommen.

(759) Elisabeth hat das Medikament, das ihr Arzt empfahl, schon lange beraten.

(760) Elisabeth hat das Medikament, das ihr Arzt empfahl, schon lange nehmen.

96

(761) Tobias hat die Blume, die seiner Nachbarin gehörte, jemals oft gegossen.

(762) Tobias hat keine Blume, die seiner Nachbarin gehörte, jemals oft gegossen.

(763) Tobias hat die Blume, die seiner Nachbarin gehörte, schon oft gegossen.

(764) Tobias hat keine Blume, die seiner Nachbarin gehörte, schon oft gegossen.

(765) Tobias hat die Blume, die seiner Nachbarin gehörte, sehr oft gegossen.

(766) Tobias hat keine Blume, die seiner Nachbarin gehörte, sehr oft gegossen.

(767) Tobias hat die Blume, die seiner Nachbarin gehörte, schon oft geheizt.

(768) Tobias hat die Blume, die seiner Nachbarin gehörte, schon oft gießen.

97

(769) Friederike hat das Projekt, das arme Länder unterstützte, jemals lange gefördert.

(770) Friederike hat kein Projekt, das arme Länder unterstützte, jemals lange gefördert.

(771) Friederike hat das Projekt, das arme Länder unterstützte, schon lange gefördert.

(772) Friederike hat kein Projekt, das arme Länder unterstützte, schon lange gefördert.

(773) Friederike hat das Projekt, das arme Länder unterstützte, sehr lange gefördert.

(774) Friederike hat kein Projekt, das arme Länder unterstützte, sehr lange gefördert.

(775) Friederike hat das Projekt, das arme Länder unterstützte, schon lange überquert.

(776) Friederike hat das Projekt, das arme Länder unterstützte, schon lange fördern.

98

(777) Bruno hat das Fest, das alte Tradition war, jemals oft gefeiert.

(778) Bruno hat kein Fest, das alte Tradition war, jemals oft gefeiert.

(779) Bruno hat das Fest, das alte Tradition war, schon oft gefeiert.

(780) Bruno hat kein Fest, das alte Tradition war, schon oft gefeiert.

(781) Bruno hat das Fest, das alte Tradition war, sehr oft gefeiert.

(782) Bruno hat kein Fest, das alte Tradition war, sehr oft gefeiert.

(783) Bruno hat das Fest, das alte Tradition war, schon oft gestrickt.

(784) Bruno hat das Fest, das alte Tradition war, schon oft feiern.

99

(785) Ariane hat den Ring, der ein Geschenk war, jemals lange besessen.

(786) Ariane hat keinen Ring, der ein Geschenk war, jemals lange besessen.

(787) Ariane hat den Ring, der ein Geschenk war, schon lange besessen.

(788) Ariane hat keinen Ring, der ein Geschenk war, schon lange besessen.

(789) Ariane hat den Ring, der ein Geschenk war, sehr lange besessen.

(790) Ariane hat keinen Ring, der ein Geschenk war, sehr lange besessen.

(791) Ariane hat den Ring, der ein Geschenk war, schon lange besucht.

(792) Ariane hat den Ring, der ein Geschenk war, schon lange besitzen.

100

(793) Luis hat den Teller, der einen Goldrand hatte, jemals oft gespült.

(794) Luis hat keinen Teller, der einen Goldrand hatte, jemals oft gespült.

(795) Luis hat den Teller, der einen Goldrand hatte, schon oft gespült.

(796) Luis hat keinen Teller, der einen Goldrand hatte, schon oft gespült.

(797) Luis hat den Teller, der einen Goldrand hatte, sehr oft gespült.

(798) Luis hat keinen Teller, der einen Goldrand hatte, sehr oft gespült.

(799) Luis hat den Teller, der einen Goldrand hatte, schon oft bereut.

(800) Luis hat den Teller, der einen Goldrand hatte, schon oft spülen.

101

(801) Magdalena hat die Brille, die dicke Gläser hatte, jemals lange benutzt.

(802) Magdalena hat keine Brille, die dicke Gläser hatte, jemals lange benutzt.

(803) Magdalena hat die Brille, die dicke Gläser hatte, schon lange benutzt.

(804) Magdalena hat keine Brille, die dicke Gläser hatte, schon lange benutzt.

(805) Magdalena hat die Brille, die dicke Gläser hatte, sehr lange benutzt.

(806) Magdalena hat keine Brille, die dicke Gläser hatte, sehr lange benutzt.

(807) Magdalena hat die Brille, die dicke Gläser hatte, schon lange getrunken.

(808) Magdalena hat die Brille, die dicke Gläser hatte, schon lange benutzen.

102

(809) Ole hat den Teppich, der bunt gemustert war, jemals oft gereinigt.

(810) Ole hat keinen Teppich, der bunt gemustert war, jemals oft gereinigt.

(811) Ole hat den Teppich, der bunt gemustert war, schon oft gereinigt.

(812) Ole hat keinen Teppich, der bunt gemustert war, schon oft gereinigt.

(813) Ole hat den Teppich, der bunt gemustert war, sehr oft gereinigt.

(814) Ole hat keinen Teppich, der bunt gemustert war, sehr oft gereinigt.

(815) Ole hat den Teppich, der bunt gemustert war, schon oft gesungen.

(816) Ole hat den Teppich, der bunt gemustert war, schon oft reinigen.

103

(817) Saskia hat die Affäre, die viele Probleme bereitete, jemals lange geführt.

(818) Saskia hat keine Affäre, die viele Probleme bereitete, jemals lange geführt.

(819) Saskia hat die Affäre, die viele Probleme bereitete, schon lange geführt.

(820) Saskia hat keine Affäre, die viele Probleme bereitete, schon lange geführt.

(821) Saskia hat die Affäre, die viele Probleme bereitete, sehr lange geführt.

(822) Saskia hat keine Affäre, die viele Probleme bereitete, sehr lange geführt.

(823) Saskia hat die Affäre, die viele Probleme bereitete, schon lange gefahren.

(824) Saskia hat die Affäre, die viele Probleme bereitete, schon lange führen.

104

(825) Josef hat das Foto, das schöne Erinnerungen weckte, jemals oft betrachtet.

(826) Josef hat kein Foto, das schöne Erinnerungen weckte, jemals oft betrachtet.

(827) Josef hat das Foto, das schöne Erinnerungen weckte, schon oft betrachtet.

(828) Josef hat kein Foto, das schöne Erinnerungen weckte, schon oft betrachtet.

(829) Josef hat das Foto, das schöne Erinnerungen weckte, sehr oft betrachtet.

(830) Josef hat kein Foto, das schöne Erinnerungen weckte, sehr oft betrachtet.

(831) Josef hat das Foto, das schöne Erinnerungen weckte, schon oft angerufen.

(832) Josef hat das Foto, das schöne Erinnerungen weckte, schon oft betrachten.

105

(833) Luca hat das Treffen, das einmal monatlich stattfand, jemals oft abgesagt.

(834) Luca hat kein Treffen, das einmal monatlich stattfand, jemals oft abgesagt.

(835) Luca hat das Treffen, das einmal monatlich stattfand, schon oft abgesagt.

(836) Luca hat kein Treffen, das einmal monatlich stattfand, schon oft abgesagt.

(837) Luca hat das Treffen, das einmal monatlich stattfand, sehr oft abgesagt.

(838) Luca hat kein Treffen, das einmal monatlich stattfand, sehr oft abgesagt.

(839) Luca hat das Treffen, das einmal monatlich stattfand, schon oft angebaut.

(840) Luca hat das Treffen, das einmal monatlich stattfand, schon oft absagen.

106

(841) Linda hat den Ausblick, der besonders schön war, jemals lange genossen.

(842) Linda hat keinen Ausblick, der besonders schön war, jemals lange genossen.

(843) Linda hat den Ausblick, der besonders schön war, schon lange genossen.

(844) Linda hat keinen Ausblick, der besonders schön war, schon lange genossen.

(845) Linda hat den Ausblick, der besonders schön war, sehr lange genossen.

(846) Linda hat keinen Ausblick, der besonders schön war, sehr lange genossen.

(847) Linda hat den Ausblick, der besonders schön war, schon lange getrunken.

(848) Linda hat den Ausblick, der besonders schön war, schon lange genießen.

107

(849) Oliver hat das Motorrad, das seinem Freund gehörte, jemals oft gefahren.

(850) Oliver hat kein Motorrad, das seinem Freund gehörte, jemals oft gefahren.

(851) Oliver hat das Motorrad, das seinem Freund gehörte, schon oft gefahren.

(852) Oliver hat kein Motorrad, das seinem Freund gehörte, schon oft gefahren.

(853) Oliver hat das Motorrad, das seinem Freund gehörte, sehr oft gefahren.

(854) Oliver hat kein Motorrad, das seinem Freund gehörte, sehr oft gefahren.

(855) Oliver hat das Motorrad, das seinem Freund gehörte, schon oft betrieben.

(856) Oliver hat das Motorrad, das seinem Freund gehörte, schon oft fahren.

108

(857) Tabea hat die Katze, die viele Mäuse fing, jemals oft gelobt.

(858) Tabea hat keine Katze, die viele Mäuse fing, jemals oft gelobt.

(859) Tabea hat die Katze, die viele Mäuse fing, schon oft gelobt.

(860) Tabea hat keine Katze, die viele Mäuse fing, schon oft gelobt.

(861) Tabea hat die Katze, die viele Mäuse fing, sehr oft gelobt.

(862) Tabea hat keine Katze, die viele Mäuse fing, sehr oft gelobt.

(863) Tabea hat die Katze, die viele Mäuse fing, schon oft vermietet.

(864) Tabea hat die Katze, die viele Mäuse fing, schon oft loben.

109

(865) Günther hat die Uhr, die goldene Zeiger hatte, jemals oft getragen.

(866) Günther hat keine Uhr, die goldene Zeiger hatte, jemals oft getragen.

(867) Günther hat die Uhr, die goldene Zeiger hatte, schon oft getragen.

(868) Günther hat keine Uhr, die goldene Zeiger hatte, schon oft getragen.

(869) Günther hat die Uhr, die goldene Zeiger hatte, sehr oft getragen.

(870) Günther hat keine Uhr, die goldene Zeiger hatte, sehr oft getragen.

(871) Günther hat die Uhr, die goldene Zeiger hatte, schon oft gebacken.

(872) Günther hat die Uhr, die goldene Zeiger hatte, schon oft tragen.

110

(873) Marie hat die Sprache, die grammatikalisch komplex war, jemals lange erlernt.

(874) Marie hat keine Sprache, die grammatikalisch komplex war, jemals lange erlernt.

(875) Marie hat die Sprache, die grammatikalisch komplex war, schon lange erlernt.

(876) Marie hat keine Sprache, die grammatikalisch komplex war, schon lange erlernt.

(877) Marie hat die Sprache, die grammatikalisch komplex war, sehr lange erlernt.

(878) Marie hat keine Sprache, die grammatikalisch komplex war, sehr lange erlernt.

(879) Marie hat die Sprache, die grammatikalisch komplex war, schon lange gekauft.

(880) Marie hat die Sprache, die grammatikalisch komplex war, schon lange erlernen.

111

(881) Karl hat den Tee, der frische Kräuter enthielt, jemals oft zubereitet.

(882) Karl hat keinen Tee, der frische Kräuter enthielt, jemals oft zubereitet.

(883) Karl hat den Tee, der frische Kräuter enthielt, schon oft zubereitet.

(884) Karl hat keinen Tee, der frische Kräuter enthielt, schon oft zubereitet.

(885) Karl hat den Tee, der frische Kräuter enthielt, sehr oft zubereitet.

(886) Karl hat keinen Tee, der frische Kräuter enthielt, sehr oft zubereitet.

(887) Karl hat den Tee, der frische Kräuter enthielt, schon oft gebügelt.

(888) Karl hat den Tee, der frische Kräuter enthielt, schon oft zubereiten.

112

(889) Andrea hat die Brille, die sie täglich trug, jemals oft geputzt.

(890) Andrea hat keine Brille, die sie täglich trug, jemals oft geputzt.

(891) Andrea hat die Brille, die sie täglich trug, schon oft geputzt.

(892) Andrea hat keine Brille, die sie täglich trug, schon oft geputzt.

(893) Andrea hat die Brille, die sie täglich trug, sehr oft geputzt.

(894) Andrea hat keine Brille, die sie täglich trug, sehr oft geputzt.

(895) Andrea hat die Brille, die sie täglich trug, schon oft unterstützt.

(896) Andrea hat die Brille, die sie täglich trug, schon oft putzen.

113

(897) Toni hat die Pizza, die dick belegt war, jemals oft bestellt.

(898) Toni hat keine Pizza, die dick belegt war, jemals oft bestellt.

(899) Toni hat die Pizza, die dick belegt war, schon oft bestellt.

(900) Toni hat keine Pizza, die dick belegt war, schon oft bestellt.

(901) Toni hat die Pizza, die dick belegt war, sehr oft bestellt.

(902) Toni hat keine Pizza, die dick belegt war, sehr oft bestellt.

(903) Toni hat die Pizza, die dick belegt war, schon oft gefördert.

(904) Toni hat die Pizza, die dick belegt war, schon oft bestellen.

114

(905) Kristin hat das Zimmer, das stark beheizt war, jemals lange gelüftet.

(906) Kristin hat kein Zimmer, das stark beheizt war, jemals lange gelüftet.

(907) Kristin hat das Zimmer, das stark beheizt war, schon lange gelüftet.

(908) Kristin hat kein Zimmer, das stark beheizt war, schon lange gelüftet.

(909) Kristin hat das Zimmer, das stark beheizt war, sehr lange gelüftet.

(910) Kristin hat kein Zimmer, das stark beheizt war, sehr lange gelüftet.

(911) Kristin hat das Zimmer, das stark beheizt war, schon lange erzählt.

(912) Kristin hat das Zimmer, das stark beheizt war, schon lange lüften.

115

(913) Martin hat die Stadt, die viele Einwohner hatte, jemals oft bereist.

(914) Martin hat keine Stadt, die viele Einwohner hatte, jemals oft bereist.

(915) Martin hat die Stadt, die viele Einwohner hatte, schon oft bereist.

(916) Martin hat keine Stadt, die viele Einwohner hatte, schon oft bereist.

(917) Martin hat die Stadt, die viele Einwohner hatte, sehr oft bereist.

(918) Martin hat keine Stadt, die viele Einwohner hatte, sehr oft bereist.

(919) Martin hat die Stadt, die viele Einwohner hatte, schon oft getragen.

(920) Martin hat die Stadt, die viele Einwohner hatte, schon oft bereisen.

116

(921) Debora hat das Foto, das qualitativ schlecht war, jemals lange bearbeitet.

(922) Debora hat kein Foto, das qualitativ schlecht war, jemals lange bearbeitet.

(923) Debora hat das Foto, das qualitativ schlecht war, schon lange bearbeitet.

(924) Debora hat kein Foto, das qualitativ schlecht war, schon lange bearbeitet.

(925) Debora hat das Foto, das qualitativ schlecht war, sehr lange bearbeitet.

(926) Debora hat kein Foto, das qualitativ schlecht war, sehr lange bearbeitet.

(927) Debora hat das Foto, das qualitativ schlecht war, schon lange erzählt.

(928) Debora hat das Foto, das qualitativ schlecht war, schon lange bearbeiten.

117

(929) Stefan hat das Kind, das sich versteckt hatte, jemals lange gesucht.

(930) Stefan hat kein Kind, das sich versteckt hatte, jemals lange gesucht.

(931) Stefan hat das Kind, das sich versteckt hatte, schon lange gesucht.

(932) Stefan hat kein Kind, das sich versteckt hatte, schon lange gesucht.

(933) Stefan hat das Kind, das sich versteckt hatte, sehr lange gesucht.

(934) Stefan hat kein Kind, das sich versteckt hatte, sehr lange gesucht.

(935) Stefan hat das Kind, das sich versteckt hatte, schon lange bewohnt.

(936) Stefan hat das Kind, das sich versteckt hatte, schon lange suchen.

118

(937) Fenja hat die Straße, die stark befahren war, jemals oft überquert.

(938) Fenja hat keine Straße, die stark befahren war, jemals oft überquert.

(939) Fenja hat die Straße, die stark befahren war, schon oft überquert.

(940) Fenja hat keine Straße, die stark befahren war, schon oft überquert.

(941) Fenja hat die Straße, die stark befahren war, sehr oft überquert.

(942) Fenja hat keine Straße, die stark befahren war, sehr oft überquert.

(943) Fenja hat die Straße, die stark befahren war, schon oft betrieben.

(944) Fenja hat die Straße, die stark befahren war, schon oft überqueren.

119

(945) Ralf hat das Haus, das seinen Eltern gehörte, jemals lange bewohnt.

(946) Ralf hat kein Haus, das seinen Eltern gehörte, jemals lange bewohnt.

(947) Ralf hat das Haus, das seinen Eltern gehörte, schon lange bewohnt.

(948) Ralf hat kein Haus, das seinen Eltern gehörte, schon lange bewohnt.

(949) Ralf hat das Haus, das seinen Eltern gehörte, sehr lange bewohnt.

(950) Ralf hat kein Haus, das seinen Eltern gehörte, sehr lange bewohnt.

(951) Ralf hat das Haus, das seinen Eltern gehörte, schon lange gelutscht.

(952) Ralf hat das Haus, das seinen Eltern gehörte, schon lange bewohnen.

120

(953) Ina hat den Urlaub, der alte Tradition war, jemals lange geplant.

(954) Ina hat keinen Urlaub, der alte Tradition war, jemals lange geplant.

(955) Ina hat den Urlaub, der alte Tradition war, schon lange geplant.

(956) Ina hat keinen Urlaub, der alte Tradition war, schon lange geplant.

(957) Ina hat den Urlaub, der alte Tradition war, sehr lange geplant.

(958) Ina hat keinen Urlaub, der alte Tradition war, sehr lange geplant.

(959) Ina hat den Urlaub, der alte Tradition war, schon lange unterrichtet.
